# Adenine Radical Cation Formation by a Ligand-Centered Excited State of an Intercalated Chromium Polypyridyl Complex Leads to Enhanced DNA Photo-oxidation

**DOI:** 10.1021/jacs.1c06658

**Published:** 2021-08-31

**Authors:** Frederico A. Baptista, Dorottya Krizsan, Mark Stitch, Igor V. Sazanovich, Ian P. Clark, Michael Towrie, Conor Long, Lara Martinez-Fernandez, Roberto Improta, Noel A. P. Kane-Maguire, John M. Kelly, Susan J. Quinn

**Affiliations:** † School of Chemistry, University College Dublin, Dublin 4, Ireland; ‡ STFC Central Laser Facility, Research Complex at Harwell, Rutherford Appleton Laboratory, Harwell Campus, Didcot, OX11 0QX, U.K.; § The School of Chemical Sciences, Dublin City University, Dublin 9, Ireland; ∥ Departamento de Química, Facultad de Ciencias and Institute for Advanced Research in Chemistry(IADCHEM) Universidad Autónoma de Madrid, Campus de Excelencia UAM-CSIC, Cantoblanco, 28049 Madrid, Spain; ⊥ Consiglio Nazionale delle Ricerche, Istituto di Biostrutture e Bioimmagini, 80136 Naples, Italy; # Department of Chemistry, Furman University, 3300 Poinsett Highway, Greenville, South Carolina 29613-1120, United States; ¶ School of Chemistry, Trinity College Dublin, The University of Dublin, Dublin 2, Ireland

## Abstract

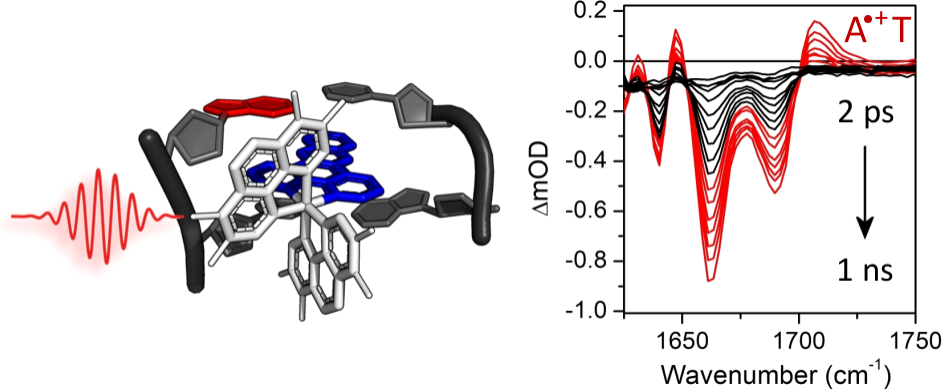

Assessment of the DNA photo-oxidation and synthetic photocatalytic activity of chromium polypyridyl complexes is dominated by consideration of their long-lived metal-centered excited states. Here we report the participation of the excited states of [Cr(TMP)_2_dppz]^3+^ (**1**) (TMP = 3,4,7,8-tetramethyl-1,10-phenanthroline; dppz = dipyrido[3,2-*a*:2′,3′-*c*]phenazine) in DNA photoreactions. The interactions of enantiomers of **1** with natural DNA or with oligodeoxynucleotides with varying AT content (0–100%) have been studied by steady state UV/visible absorption and luminescence spectroscopic methods, and the emission of **1** is found to be quenched in all systems. The time-resolved infrared (TRIR) and visible absorption spectra (TA) of **1** following excitation in the region between 350 to 400 nm reveal the presence of relatively long-lived dppz-centered states which eventually yield the emissive metal-centered state. The dppz-localized states are fully quenched when bound by GC base pairs and partially so in the presence of an AT base-pair system to generate purine radical cations. The sensitized formation of the adenine radical cation species (**A^•+^T**) is identified by assigning the TRIR spectra with help of DFT calculations. In natural DNA and oligodeoxynucleotides containing a mixture of AT and GC of base pairs, the observed time-resolved spectra are consistent with eventual photo-oxidation occurring predominantly at guanine through hole migration between base pairs. The combined targeting of purines leads to enhanced photo-oxidation of guanine. These results show that DNA photo-oxidation by the intercalated **1**, which locates the dppz in contact with the target purines, is dominated by the LC centered excited state. This work has implications for future phototherapeutics and photocatalysis.

## Introduction

The role of transition metal complexes in causing photo-oxidation in DNA is well established, and they are increasingly being considered as an alternative to porphyrins for light-activated therapeutic applications.^1−6^ Many (perhaps most) of these complexes are expected to generate singlet oxygen which can then attack cellular biomolecules, including nucleic acids.^1−6^ However, another possible mechanism, which has become apparent over the past few years, is the direct oxidation of guanine and DNA damage by photoinduced one-electron abstraction by the excited state of a proximal metal complex.^7−11^ In order to facilitate such a reaction with DNA one can incorporate ligands that will cause the complex to bind strongly to DNA with well-defined geometry. This can be achieved using complexes containing a ligand which can intercalate between the base pairs of DNA. One class of such metal complexes, which has been much studied over recent years, is that containing (dipyrido[3,2-*a*:2′,3′-*c*]phenazine), (dppz) such as [Ru(phen)_2_(dppz)]^2+^ (phen = 1,10 phenanthroline).^12^ An additional feature of these complexes is that they exist as enantiomers,^13,14^ which can show different DNA-binding and photophysical properties. [Ru(phen)_2_(dppz)]^2+^ is itself unable to photo-oxidize DNA, but it is possible to increase the oxidizing power of the excited state by changing the ligand (for example replacing phen by 1,4,5,8-tetraazaphenanthrene (TAP)^8^) or by replacing the metal (e.g., with chromium).

Exploration of the photochemistry and photophysics of Cr(III) systems is second only to ruthenium(II) among the transition metals.^15−22^ Recently the potential to tune their photophysical properties to enhance their photonic and photocatalytic behavior has attracted considerable attention.^23−29^ Upon photoexcitation chromium complexes are known to be more strongly oxidizing than their ruthenium counterparts, and in the case of Cr(diimine)_3_^3+^ complexes the oxidizing power of the relatively long-lived (μs) lowest doublet metal-centered ^2^E/^2^T_1_ (^2^MC) excited state is of the order of 1.4 V v SHE.^18−20^ Furthermore, in contrast to the Ru(diimine)_3_^2+^ complexes, whose excited state chemistry is dominated by metal-to-ligand-charge-transfer (MLCT) processes, other excited states, including ligand-centered (LC) states, are available to chromium systems. In addition, such LC states are expected to be of significantly higher energy than the long-lived LC ππ* triplet state observed for ruthenium complexes containing the extended dppn (benzo[*i*]dipyrido[3,2-*a*:2′,3′-*c*]phenazine) ligand, which damages DNA through sensitized singlet oxygen formation.^30,31^

It has been shown by our groups that [Cr(phen)_2_(dppz)]^3+^ and related complexes bind strongly to double-stranded DNA and that this is accompanied by the quenching of the emission of the Cr-complex, which is consistent with its excited state being capable of oxidizing guanine.^32−37^ It was also found that the luminescence of [Cr(phen)_2_(dppz)]^3+^ is dynamically quenched by 5′-guanosine monophosphate (GMP) with a rate constant close to that of diffusion control, while 5′-adenosine monophosphate (AMP) was almost 3 orders of magnitude less effective.^34^ Though the behavior with GMP is entirely consistent with quenching via electron transfer, no evidence of the reduced complex could be found using nanosecond flash photolysis,^33,34^ suggesting that the back reaction with the oxidized GMP must also be very fast.

Time-resolved infrared spectroscopic (TRIR) measurements have been shown to be particularly valuable for studying transient species from intercalator-DNA systems, as the spectra can provide information not only about the intercalator but also about the behavior of the host DNA site, which is revealed by “bleach” bands in the 1600–1750 cm^–1^ region.^10,38−42^ TRIR also allows detection of the guanine radical cation, which absorbs at 1700 cm^–1^.^7,43,44^ Such data are not readily obtainable by visible transient absorption (TA), as generally DNA transient species only absorb weakly at wavelengths >400 nm.^40^ We have extensively studied the photo-oxidizing complex [Ru(TAP)_2_dppz]^2+^ (*E*^0^(*Ru^2+^/Ru^+^), = 1.44 V vs SHE) using picosecond TA and TRIR^40^ and have correlated the photophysical properties in solution to structural data obtained by X-ray studies on the DNA bound systems.^40,45,46^ More recently we investigated the electron transfer reaction of racemic *rac*-[Cr(phen)_2_(dppz)]^3+^ bound to guanine-containing oligodeoxynucleotides (ODNs) using these techniques and discovered that in those cases the excited state was quenched with rates faster than 3 ps.^35^

The unsubstituted TAP and phen allow deep intercalation of the dppz ligand, and X-ray crystallography reveals that the isostructural complexes bind in a similar fashion.^40^ However, the presence of bulky ancillary ligands has been found to influence the DNA binding site. In particular, dppz complexes with 3,4,7,8 tetramethyl-1,10-phenanthroline (TMP) as an ancillary ligand have been found to show a preference for AT DNA.^32,36,47,48^ This was reported for the Cr(TMP)_2_dppz^3+^ (**1**)^32^ and related complexes^36^ and subsequently for the Ru(TMP)_2_dppz system.^47^ In the case of the ruthenium system theoretical studies showed preference for binding from the minor groove to d(ATATAT)_2_ over d(GCGCGC)_2_.^48^ Interestingly, Barton and co-workers showed that the presence of the bulky ancillary ligands demonstrated a preference for mis-match DNA.^49^

**Figure 1:**
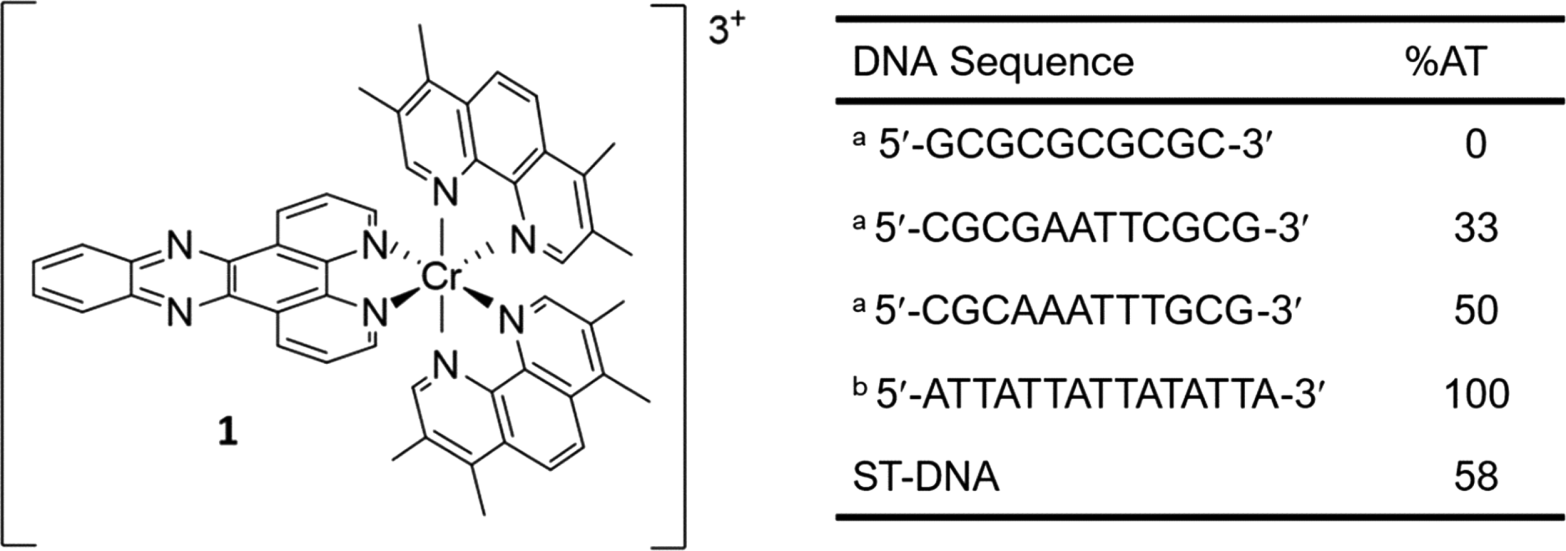
Structure of [Cr(TMP)_2_dppz]^3+^ complex (**1**) together with double stranded DNA systems ^a^self-complementary and ^b^with the complementary strand 5′-TAATAATAATATAAT-3′.

In the ultrafast spectroscopic study below we examine the photo-oxidation of DNA by each of the enantiomers of [Cr(TMP)_2_dppz]^3+^ and particularly of the lambda enantiomer which the Kane-Maguire/Wheeler group has previously reported demonstrates a strong preferential binding to DNA and most prominently for AT-rich polynucleotides.^32^ This complex therefore has the potential to target AT-rich sites, such as the TATA box.^50^ Here, we report the steady state and ps-/ns-transient spectroscopic studies of the lambda and delta enantiomers of [Cr(TMP)_2_(dppz)]^3+^ bound to four ODN systems, namely the self-complementary sequences d(GCGCGCGCGC)_2_, d(CGCGAATTCGCG)_2_, d(CGCAAATTTGCG)_2_, and d(ATTATTATTATATTA)/d(TAATATAATAATAAT) duplex (AT DNA), as well as to natural (salmon testes, ST-DNA) DNA, Figure 1. Together the AT base-pair content of these systems range from 0% to 100%. Significantly these studies reveal a role for upper (dppz-localized) excited states in the oxidation of the adenine (as well as the guanine) nucleobases forming the radical cations, which are identified by TRIR. To the best of our knowledge this is the first case where the LC state of a Cr(diimine)_3_^3+^ complex participates in direct electron transfer with purine bases and the first observation of an intercalating metal complex sensitizing adenosine oxidation in DNA.

## Results

### Steady State Spectroscopic Study of DNA Binding

*rac*-[Cr(TMP)_2_(dppz)]Cl_3_ (**1**) was prepared as previously reported,^51^ and its enantiomers were resolved by passing the racemic compound through a C25-sephadex column eluted with a (−)-*O*,*O*′-dibenzoyl-l-tartrate mobile phase. The UV–visible and circular dichroism (CD) spectra of the resolved samples (**Λ-**1****; 76.5% and Δ-**1**; 79.0%) are shown in Figure S1 and indicate details summarized in Table S1. The near-UV region of the spectrum (300–380 nm) is dominated by a structured band, showing peaks at 360 and 377 nm, which may be assigned to a dppz ligand-centered (LC) (ππ*) transition.^32^ In the visible region weak peaks at approximately 425 and 457 nm are ascribed to the metal-centered (d–d) (^4^A_2_ → ^4^T_2_ (O_h_) transitions. Excitation of the complex in the near UV or visible region results in a sharp emission at 737 nm, assigned to the ^2^E/^2^T_1_ → ^4^A_2_ transition from a doublet metal-centered (^2^MC) state (Figure S2).

**Figure 2:**
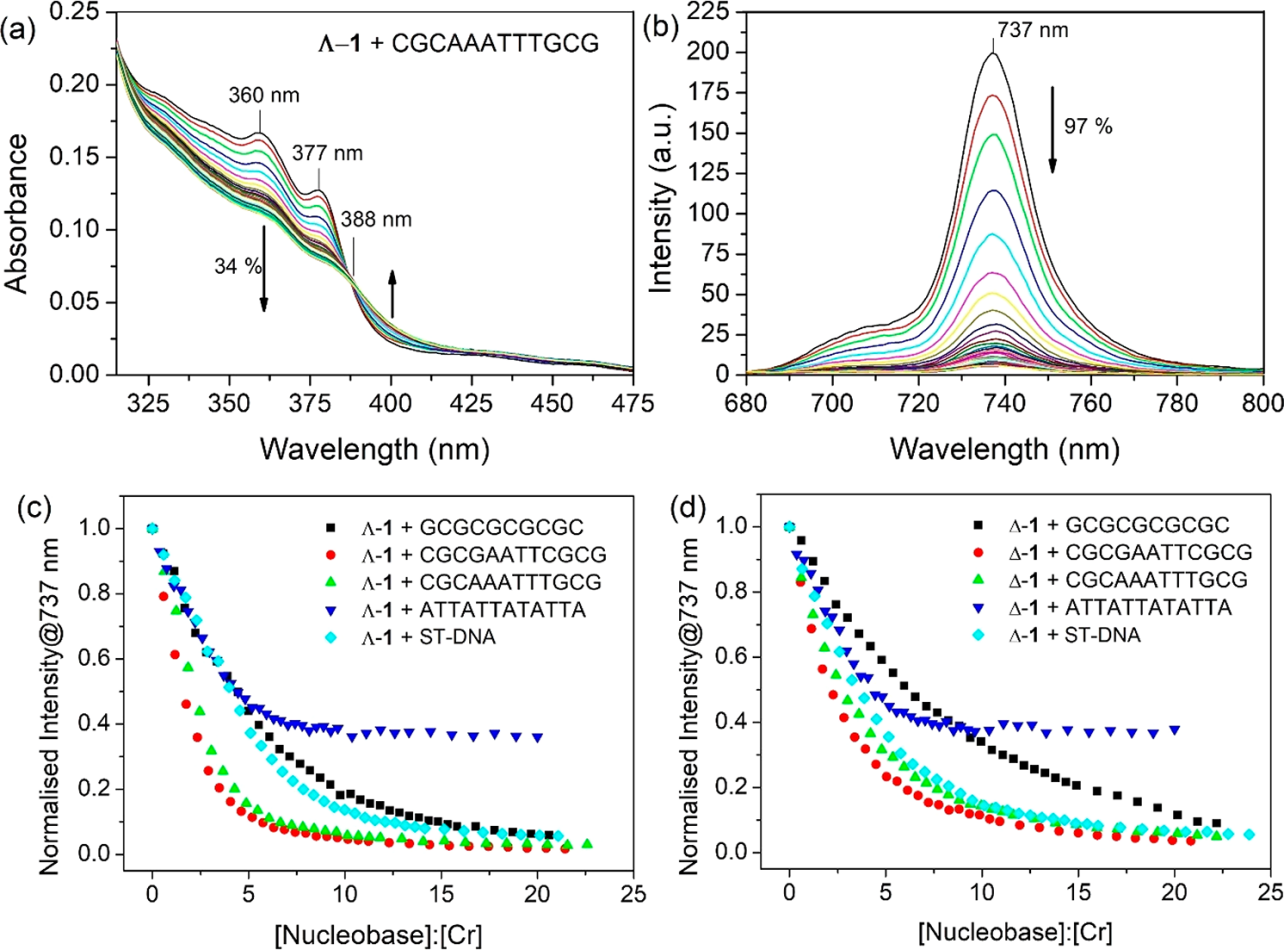
(a) UV–vis spectra and (b) emission of 10 μM **Λ-1** increasing concentrations of CGCAAATTTGCG (0–230 μM Nucleobase) in the presence of 50 mM potassium phosphate buffer, pH 7. Normalized intensity at 737 nm for (c) **Λ-1** and (d) **Δ-1** in the presence of the different DNA sequences (Excitation at 373 nm).

The emission of **1** is found to be quenched in the presence of GMP. Notably, 5′-deoxyadenosine monophosphate (dAMP) is also observed to quench the emission but significantly less effectively. Stern–Volmer plots show that quenching occurs for GMP with a *k*_q_ value of 1.7 × 10^9^ dm^3^ mol^–1^ s^–1^, close to the diffusion limit, while a lower rate constant of 4.2 × 10^6^ dm^3^ mol^–1^ s^–1^ is observed for dAMP (see Figure S3). This result is similar to that observed for the Cr(phen)_2_dppz complex.^34^ Titrations of the enantiomers with aliquots of the various DNA/ODN samples shown in Figure 1 were performed and monitored by UV/visible and emission spectroscopy (Figures 2 and S4–S5). In all cases a diminution of the absorbance of the peaks at 360 and 377 nm (associated with the ππ* transitions of the dppz ligand) was observed, with a well-defined isosbestic point at 388 nm and enhanced absorption at wavelengths beyond that (Figure S4). Such behavior is consistent with intercalation of the dppz ligand.^32,33^

Monitoring the emission demonstrates that in each case the emission at 737 nm is at least partially quenched upon addition of aliquots of the DNA or ODN (Figure 2c–d and Figure S5). In contrast to the dynamic quenching observed in the presence of GMP and dAMP, it is expected that for the DNA or ODN systems the process will involve static quenching as was previously shown for the analogous [Cr(phen)_2_dppz^3+^].^34^ Plots of the absorbance (Figure 2a, Figure S4) and emission (Figure 2b, Figure S5) changes reveal that in all cases the effect is larger for the lambda complex than for its delta enantiomer consistent with it binding more strongly. Binding constants derived from the emission data using the Bard equation^52^ are given in Table S2, and the fitted data are shown in Figure S6. The comparison of the emission titrations (Figure 2c–d) indicates that the emission quenching at [Nucleobase]/[Cr]>20 is high in all cases (>90%) for guanine-containing DNAs. Importantly, significant quenching (ca. 60%) is observed for the d(ATTATTATTATATTA)/(TAATATAATAATAAT) sequence, indicating that the excited state can be deactivated by adenine. Differences in the binding behavior of the Λ and Δ enantiomer are most apparent for the CGCGAATTCGCG, CGCAAATTTGCG, and ST-DNA DNA systems with the largest effect found for CGCAAATTTCGC (see Figure S4).

### ps- and ns-Transient Absorption (TA) of [Cr(TMP)_2_(dppz)]^3+^ in Solution

**Figure 3:**
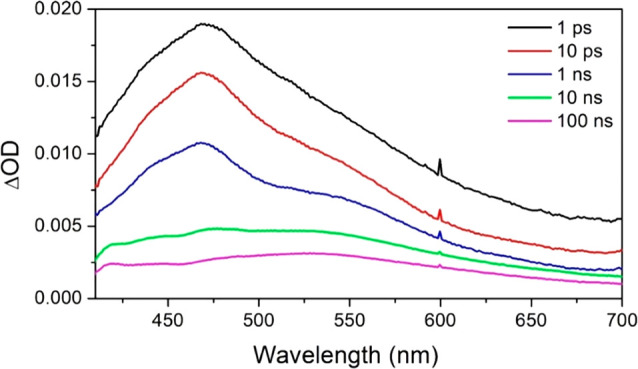
ps-ns-TA spectrum of 1 mM of ***rac*****-1** in the presence of 50 mM of sodium phosphate buffer in D_2_O (excitation 373 nm (ps)/355 nm (ns)).

To understand the mechanisms for quenching of the excited state of [Cr(TMP)_2_(dppz)]^3+^ by the various DNA systems, we first investigated the behavior of [Cr(TMP)_2_(dppz)]^3+^ in D_2_O or D_2_O/buffer solutions. Figure 3 (and Figure S7) shows the results of TA experiments, monitoring on both picosecond and nanosecond time scales following excitation at 355 or 373 nm. Similar results were obtained in both D_2_O and D_2_O/50 mM potassium phosphate buffer. It may be observed that 1 ps after excitation a new species is present which shows maximum absorption at 469 nm and several prominent shoulders. This transient spectrum is similar to that previously observed for dppz itself^53^ and for [Re(CO)_3_py(dppz)]^+^^54^ and assigned to a ππ* excited state. It is therefore consistent with this initially formed species from **1** being a ligand-centered (LC) excited state. The absorbance at 469 nm then partially decays which can be modeled by biexponential kinetics with lifetimes of 4.3 ps (28%) and 460 ps (23%) with the remainder decaying at longer times. We propose that the first species observed is the LC-state [^4^Cr(TMP)_2_^1^dppz]^3+^* and that this species then decays with a lifetime of 4.3 ps via intersystem crossing to [^4^Cr(TMP)_2_^3^dppz]^3+^*. TD-DFT simulations (see Supporting Information (SI) for details), which we have shown to be reliable for similar metal complexes,^41,55^ were carried out for this latter species and show indeed that it is predicted to have a strong transient absorption band in this spectral region (Figure S8).

Probing **1** on the nanosecond time scale (up to 500 ns) reveals a major change as the species showing maximum absorption at 469 nm decays with a lifetime of 4 ns and a new species possessing a broad absorption band with a maximum at ca. 530 nm is formed (see Figure 3). This latter species decays away only very slowly indicating that it has a lifetime greater than 500 ns, as expected for an emissive ^2^MC excited state.^34,35^ It is clear from these ps and ns TA experiments that excitation in the range 350–400 nm produces LC-species with rather long lifetimes, and it is intriguing to consider whether they might react when bound to DNA, which could be most readily observed by TRIR.

### Time-Resolved Infrared (TRIR) Studies of [Cr(TMP)_2_(dppz)]^3+^ in Solution

**Figure 4:**
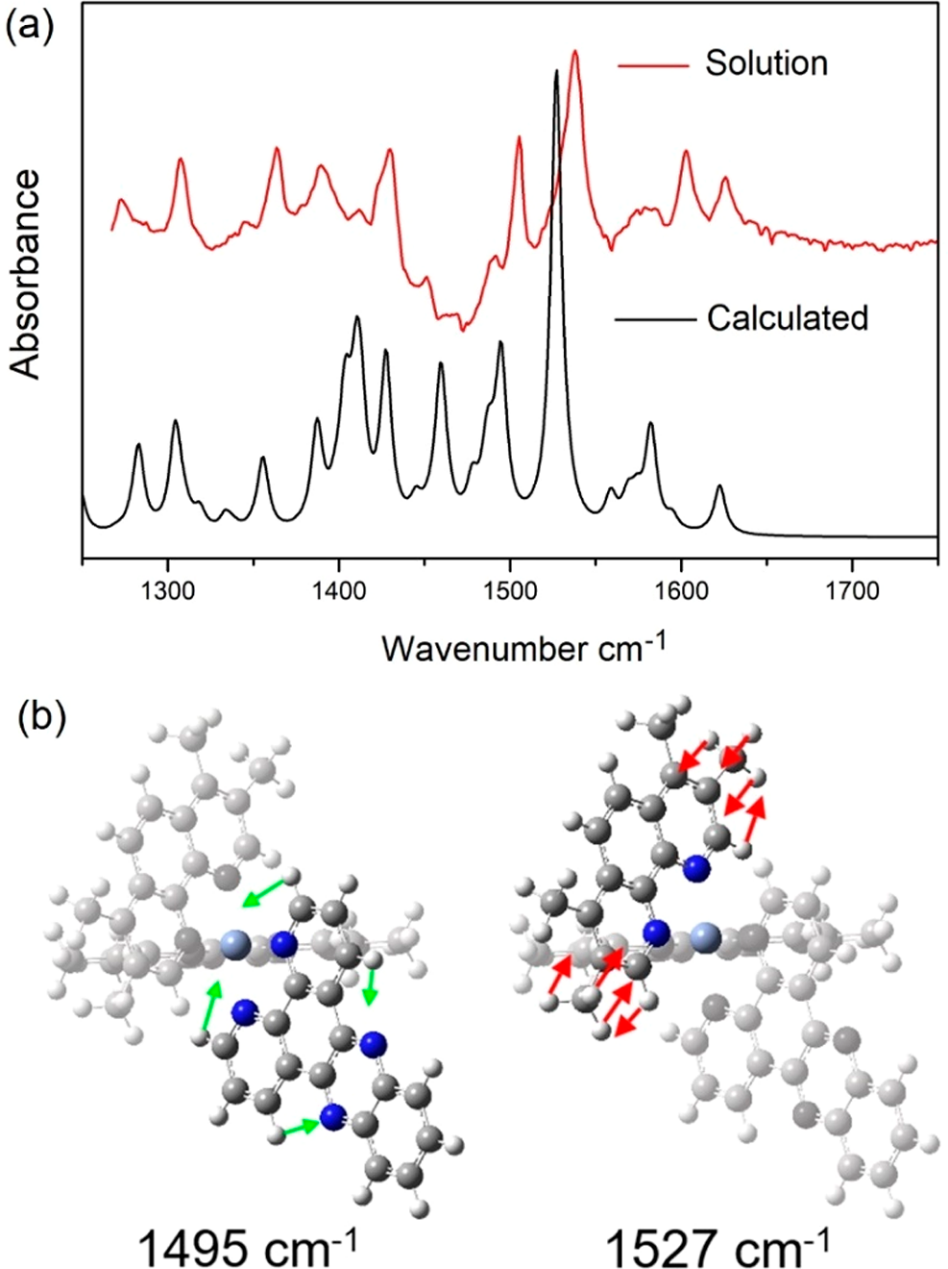
(a) Comparison of the calculated (B3LYP/LanL2DZ; see SI for further details) and experimental infrared spectra of **1**. *Absence of the band at 1460 cm^–1^ in the ground state FTIR arises due to imperfect blank against D_2_O solution containing contributions from HOD. (b) Highlighted key simulated dppz and TMP ground state vibrations.

ps- and ns-TRIR experiments were first carried out on the [Cr(TMP)_2_(dppz)]^3+^ solutions under comparable conditions to those used for the TA (see SI). Ground state IR (FTIR) spectrum of **1** recorded in D_2_O is highly structured arising from vibrations on the TMP and dppz ligands (Figure 4). This solution spectrum is found to be in excellent agreement with the spectrum simulated using unrestricted DFT calculations (see Experimental Section). These simulations were used to assign vibrations of the complex (Table S3, Figure S9), including those localized on the two TMP ligands (observed to occur at 1635 cm^–1^ (CH_3_ phen coupled) and 1540 cm^–1^ (C=N phen)) and dppz based vibrations at 1495 and 1505 cm^–1^.

**Figure 5:**
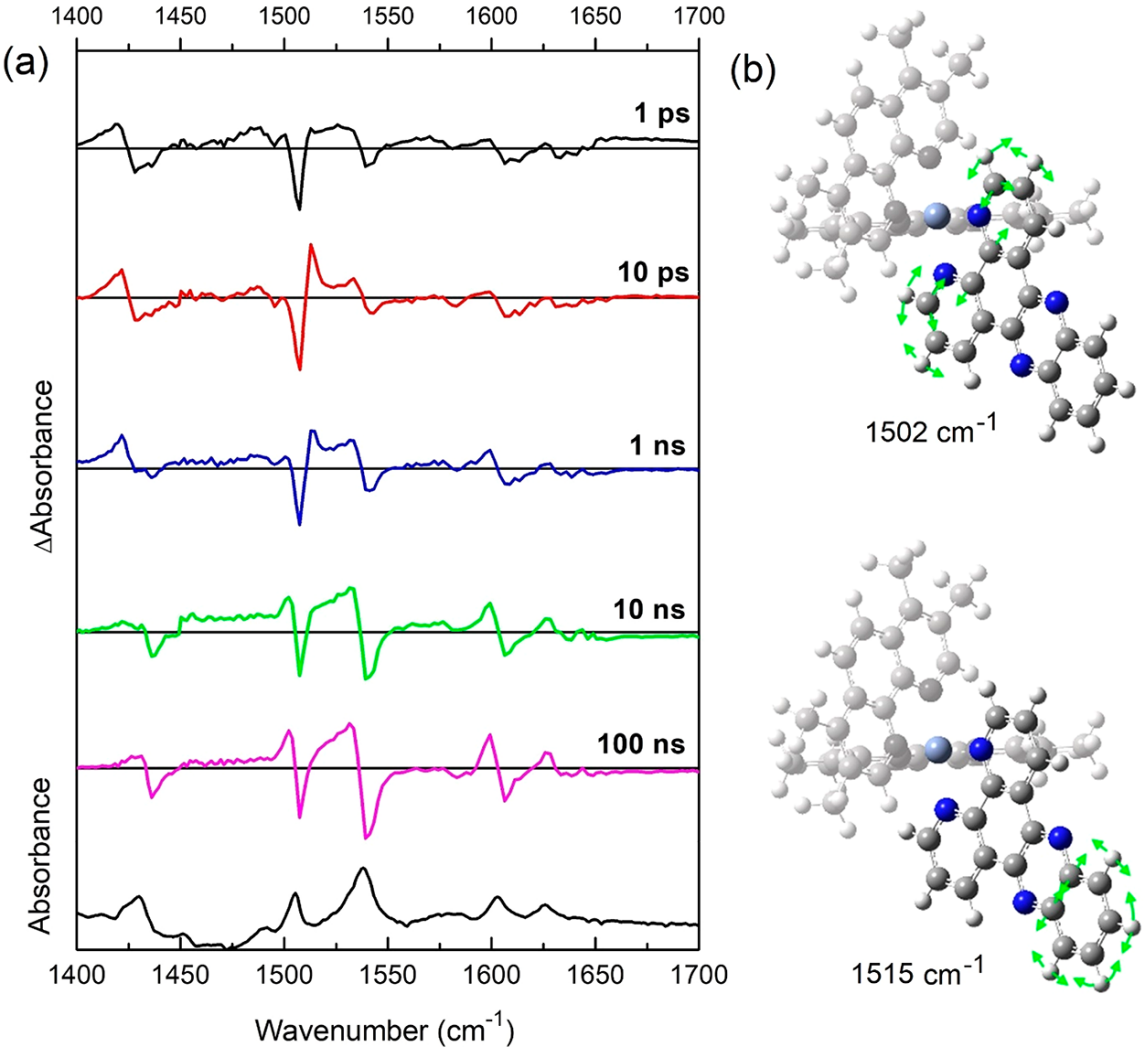
(a) ps-ns-TRIR spectrum of 1 mM of ***rac*****-[Cr(TMP)**_**2**_**dppz]**^**3+**^ in the presence of 50 mM of sodium phosphate buffer in D_2_O upon 400 nm (5 kHz, 1 μJ) excitation. (b) Calculated (see SI for details) dppz LC vibrations of the [^4^Cr(TMP)_2_(^3^dppz)]^3+^ excited state species.

TRIR spectra commonly show characteristic positive absorption bands of the transient species and also negative “bleach” bands due to depletion of the ground state. Spectra at selected times following 150 fs 400 nm excitation of [Cr(TMP)_2_(dppz)]^3+^ in D_2_O phosphate buffer solution are shown in Figure 5. Between 1 and 10 ps (where a significant diminution of the TA band at 469 nm was observed) a strong growth of a sharp band at 1512 cm^–1^ is seen. Subsequently, over the next nanosecond this band partially diminishes, and simultaneously, transient bands at 1502 and 1531 cm^–1^ are strengthened and the bleach at 1539 cm^–1^ associated with TMP vibrations increases. By 100 ns, when we anticipate that the ^2^MC is formed, one can see that the bleach pattern is in good agreement with that of the FTIR absorption bands (Figure 5a). Each of these bands are accompanied at lower wavenumber by absorption bands which we assign to the ^2^MC state. The spectra at shorter times, especially in the 1500–1550 cm^–1^ region, provide further insights into the complex interconversion of species eventually leading to this low-lying state. In the spectrum at 1 ps it may be noted that the bleaching of the vibration at 1505 cm^–1^ (dppz) is much stronger than that at 1539 cm^–1^ (TMP) in contrast to what is found in the FTIR. This indicates that the frequency of this TMP-localized vibration is essentially unaffected upon formation of the excited state, as expected for a purely dppz-localized excited state. The subsequent increase in the bleaching intensity of this band is consistent with the formation of the MC state and the consequent changes in the bonding to the TMP. Kinetic analysis of the grow-in of the transient at 1530 cm^–1^ and the bleach 1539 cm^–1^ reveals a process on the 5 ns time scale (Figure S10), which is in good agreement with the TA (Figure 4). As noted above from the TA measurements we propose that initial excitation leads to the formation of the singlet dppz-centered excited state [^4^Cr(TMP)_2_(^1^dppz)]^3+^, which leads to the formation of a [^4^Cr(TMP)_2_(^3^dppz)]^3+^ (5 ps) (eq 1). DFT calculations predict that this latter species shows the characteristic sharp band 10 cm^–1^ higher than the ground state vibration found experimentally at 1505 cm^–1^ (Figures 5b, S11, and S12) A further currently unassigned species is then formed which then yields the long-lived ^2^MC state (eq 2).
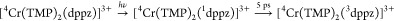
1
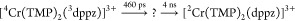
2

### Time-Resolved DNA Studies

#### ps-Time-Resolved Infrared (TRIR) of **1** in a GC ODN

To investigate the photochemical and photophysical processes occurring when **1** is excited when bound to DNA, the TRIR spectra of a series of defined-sequence ODNs (Figure 1) were next recorded. In general, the results involving the lambda and delta enantiomers are similar, and we will focus initially on the lambda species.

**Figure 6:**
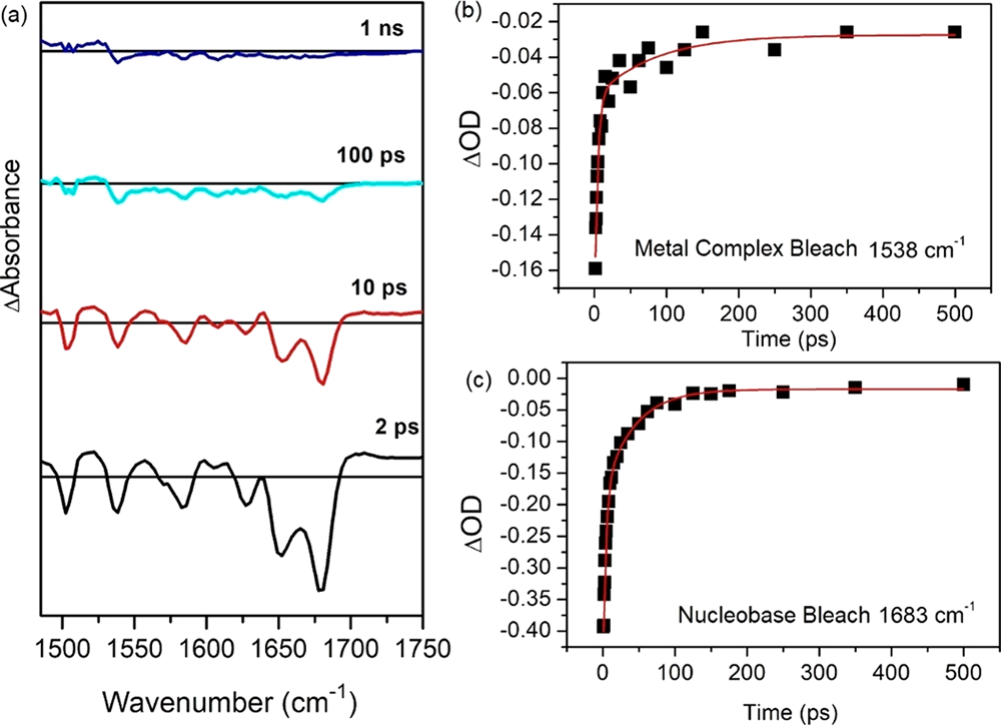
(a) ps-ns-TRIR spectrum of 1 mM of Λ-**1** in the presence of 25 mM nucleotide of d(GCGCGCGCGC)_2_ in 50 mM of sodium phosphate buffer in D_2_O (λ_exc_ = 373 nm, 5 kHz, 1 μJ). Kinetics showing recovery of (b) the metal complex bleach at 1538 cm^–1^ and (c) guanine carbonyl at 1683 cm^–1^.

First, we consider the case when the lambda enantiomer is bound to an ODN possessing only G and C nucleobases - d(GCGCGCGCGC)_2_. As noted above, the emission of **1** is strongly quenched in the presence of d(GCGCGCGCGC)_2_ at high [Nucleobase]/Cr ratios (Figure 2c). TRIR spectra (at [Nucleobase]/Cr = 20, where the quenching is >95%) recorded from 1 ps to 2.8 ns after laser excitation (50 fs; 373 nm) are shown in Figure 6a. These spectra show bleach bands characteristic of the metal complex, which are dwarfed by those corresponding to the carbonyl vibrations of the C (1650 cm^–1^) and G (1680 cm^–1^) nucleobases. The bleaching of the nucleobase bands is similar to that found for the oxidation of guanine in base-paired ODNs, as reported with other DNA-intercalated dppz complexes.^40,41^ There is also a weak absorption band at *ca*. 1700 cm^–1^, where it is predicted from DFT studies that the guanine radical cation (G^•+^) absorbs.^43,56−58^ It may also be noted that the pattern of the bleaches for the metal complex is quite different from that found for **1** in solution. This suggests that the excited state initially formed by the excitation pulse ([^4^Cr(TMP)_2_(^1^dppz)]^3+^) reacts with the ODN on a subpicosecond time scale, i.e. faster than the formation of the triplet state [^4^Cr(TMP)_2_(^3^dppz)]^3+^. We propose that the species observed on the picosecond time scale is [^4^Cr(TMP)_2_**(dppz**^**•–**^**)]**^**2+**^**/(G**^**•+**^**C)** contact ion pair. In contrast to **1** in solution, its characteristic transient and bleach bands are found to decay and recover rapidly without evolution of the spectral structure. The bleach bands of the Cr-complex recover at the same rate as those of the nucleobase which follow biphasic kinetics lifetimes of 3.7 ps (63%) and 42 ps (37%) (see Figure 6b–c). It may be noted that at 1 ns the signal at 1505 cm^–1^and 1540 cm^–1^ is very weak indicating the almost total suppression of the ^2^MC state, or other excited states/transient species. We ascribe this to back electron transfer reforming the ground state (eq 4).
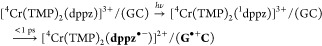
3
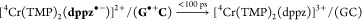
4

### ps-Time-Resolved Infrared (TRIR) of 1 in an AT ODN

**Figure 7:**
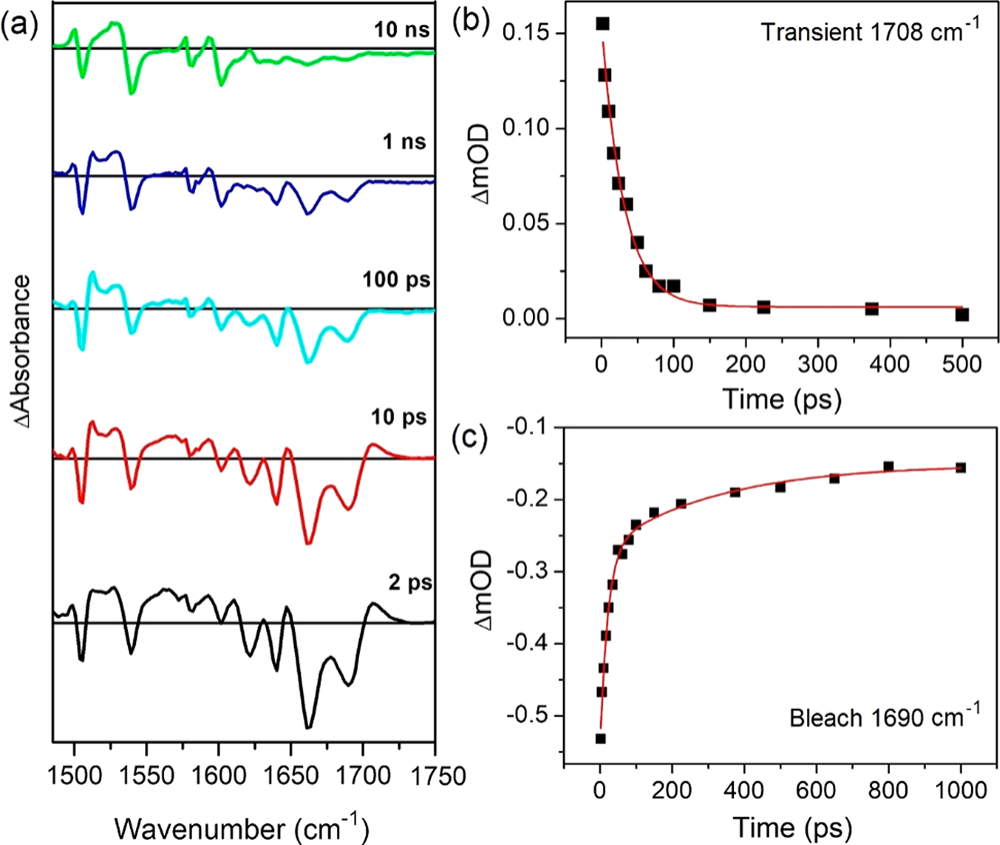
ps-ns-TRIR (400 nm excitation) spectrum of 1 mM of Λ-[Cr(TMP)_2_dppz]^3+^ in the presence of 25 mM nucleobase of (a) d(ATTATTATTATATTA)/d(TAATATAATAATAAT) duplex in 50 mM of sodium phosphate buffer in D_2_O. Kinetics showing recovery of (b) the transient at 1708 cm^–1^ and (d) bleach at 1690 cm^–1^.

Considering the TRIR of **1** bound to the d(ATTATTATTATATTA)/d(TAATATAATAATAAT) (Figure 7), one can see that as with the complex bound to d(GCGCGCGCGC)_2_ the spectrum is dominated by the bleach bands for the ODN (in this case, at 1640 cm^–1^ (T), 1622 cm^–1^ (A), 1662 cm^–1^ (T), 1690 cm^–1^ (T)) and of the complex (at 1505 and 1539 cm^–1^).^59,60^ However, in contrast to what is found with d(GCGCGCGCGC)_2_ there is also a transient species showing a very strong absorption at 1708 cm^–1^. This decays away rapidly (by first-order kinetics τ = 27 ± 2 ps) and is accompanied by partial recovery of the nucleobase bleaches (τ = 26 ± 2 ps (54%) at 1690 cm^–1^). The recovery of the bleaches continues within the nanosecond time domain (1.5 ± 0.2 ns (33%)) and the residual portion at longer times (100 ns).

Examining the bleaches due to the complex at 1505 and 1539 cm^–1^, it may be noted that unlike what was observed with d(GC)_5_ these bleaches only recover slightly in the picosecond range, indicating that most of the ground state is still not reformed by 1 ns. However, the ratio of the absorbance of the bleach bands (*A*_1505_/*A*_1539_) changes significantly during the period, increasing up to 100 ps and then gradually decreasing up to 3 ns. At short times (<10 ps) broad transient absorption is also seen at 1565 and 1525 cm^–1^. After these bands have decayed a sharp absorption band at 1515 cm^–1^ is present. This feature is similar to that observed in the TRIR of the complex itself in solution and assigned to [^4^Cr(TMP)_2_(^3^dppz)]^3+^. At much longer times (e.g., 10 ns) the spectrum shows features, which are similar to those of the ^2^MC state formed in solution (Figure 5). At this stage the bleach bands in the nucleobase region are very weak, consistent with the ^2^MC state causing very little perturbation of these DNA-localized bands.

The appearance of a reversible transient band at 1708 cm^–1^ is consistent with the formation of a short-lived DNA photoproduct, and in the absence of guanine, we assign this to the formation of A^•+^T species, as adenine is the next most readily oxidized nucleobase.^61,62^

### Characterizing the Spectral Signatures of A^•+^T

**Figure 8:**
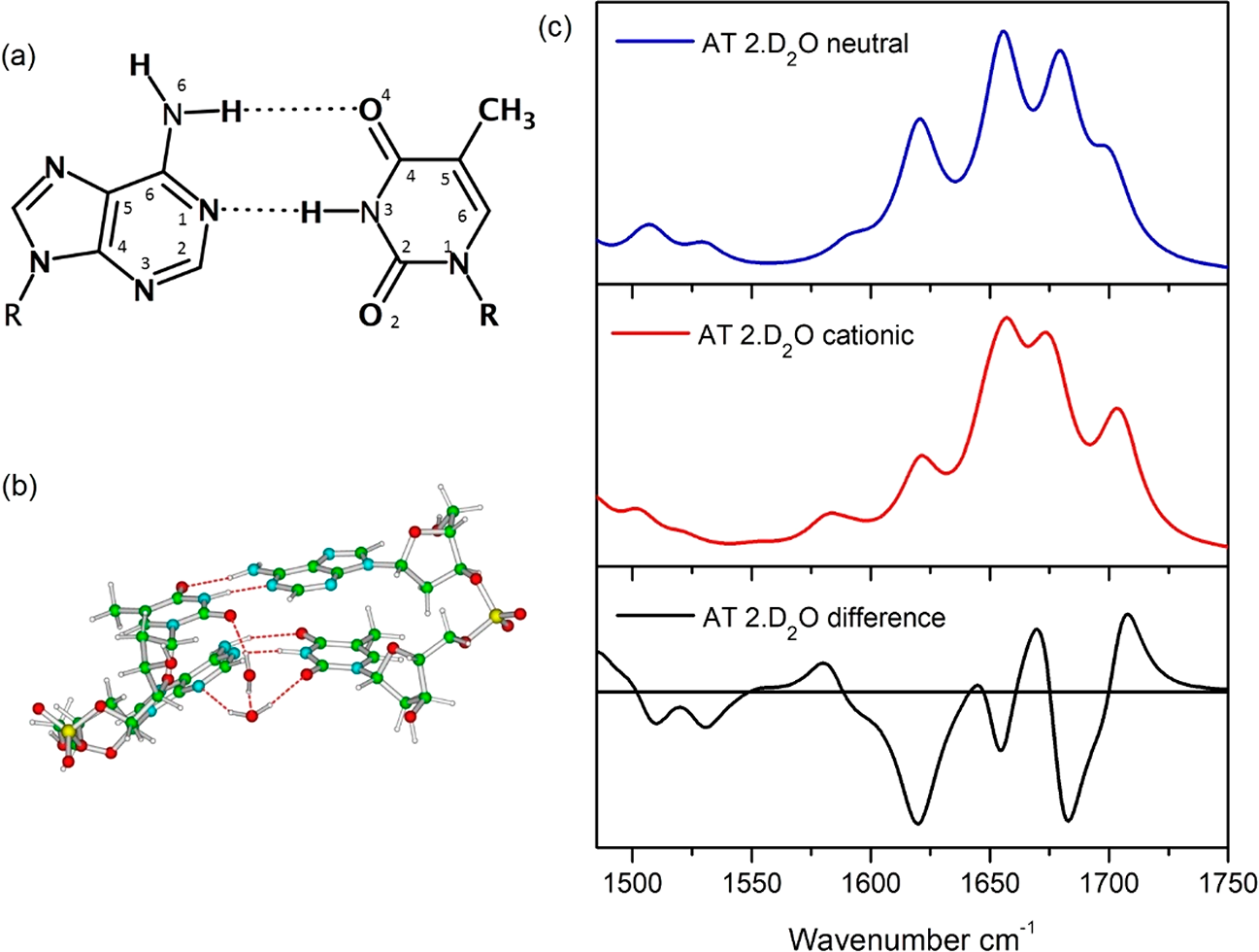
(a) A:T Watson Crick base pair. (b) Schematic description of the computational AT·2 D_2_O system. (c) Computed difference spectra between the cationic and the neutral forms of AT·2 D_2_O system. PCM/M052X/6-31G(d) calculations. Each transition has been broadened with a Gaussian with h.w.h.m. = 10 cm^–1^. Harmonic spectra have been uniformly scaled by 0.955.

In order to confirm our hypothesis for the formation of the A^•+^T species, simulated IR spectra for portions of the AT-ODN (namely TpdApT (TAT), TpdAdA (TAA), dApTpT (ATT)) containing the adenine cation (Ade^•+^) in a (dApT) dinucleotide fragment (hereafter AT), were computed. In each case the simulated IR spectra of Watson–Crick (WC) base-paired duplexes formed by these species in the neutral and in the cationic form were produced (see Figure 8). These calculations were performed at the PCM/M052X/631G(d) level (SI and methods for further details). In addition, the inclusion of explicit D_2_O molecules and Na^+^ counterions were also considered. The number of Na^+^ ions was selected in order to balance the negative charges of the phosphate backbone, considering that a detailed characterization of the ions location around DNA would require an extensive sampling by Molecular Dynamics simulations and it is well beyond the scope of this study. For what concerns D_2_O molecules, we selected those we expect to be more tightly bound to the bases, i.e. the two molecules forming hydrogen bonding (HB) with atoms not directly involved in the WC pairs (C=O2 of Thymine, Scheme S1). The calculated IR spectra of the neutral DNA bands in the 1600–1700 cm^–1^ region are in close agreement with the previously assigned experimental values (Table S4).^59,60^ These calculations reveal that the presence of Ade^•+^ leads to a noticeable perturbation of the local DNA structure, especially of the WC pairing. The HB interaction with the C4=O4 WC-paired thymine group becomes stronger, due to the larger HB accepting power of the electron deficient Ade^•+^ cation. Consequently, the thymine C4=O4 bond length increases and the associated stretching frequencies red-shifts. This also results in a weakening of the adenine “coupling” to the C2=O2 thymine bond accompanied by a slight decrease in the bond, causing the stretching frequencies to blue-shift and becoming more intense. Overall, the C=O stretching mode is more localized on a single C=O group. Simultaneously, the ring-stretching mode of Ade^•+^ red-shifts with respect to the Ade and the role of the N–H_2_ scissoring modes increases, since this bond acquires a partial double bond character (the delocalization of the amino lone pair in the electron deficient ring is favored).

The computed difference (TRIR) spectrum obtained for the AT·2D_2_O system is in good agreement with the experimental one, with the appearance of bleaches associated with both the adenine and thymine bases and a transient band at ca. > 1700 cm^–1^ (see Figure 8c and Figure S13). There are bleaches at 1620 cm^–1^ (adenine), 1654 cm^–1^ (thymine carbonyl), 1684 cm^–1^ and notably the transient band at 1708 cm^–1^, which is in excellent agreement with the experimental observation in Figure 7. The explicit D_2_O molecules simply red-shift (from ∼1720 cm^–1^ to ∼1700 cm^–1^) the peaks associated with the thymine C=O groups not involved in the interstrand HB interactions, supporting the reliability of the predictions obtained by including solvent effect exclusively at the PCM level. Importantly, it is confirmed that the presence of the band at 1708 cm^–1^ is due to the influence of the adenine cation on the WC paired thymine base. As detailed in Figures S13–S16, the difference spectra computed for the different trinucleotide examined are similar to those depicted in Figure 8 and a similar spectrum is obtained on the simple Ade-Thy WC dimer, confirming our assignment of the spectra. Analogously, the computed spectra exhibit a very small dependence on the inclusion of Na^+^ counterions (compare Figures 8 and S13), especially in the AT dinucleotide model, suggesting that the computed difference spectra are dominated by more “local” interactions (see also our results on the simple WC dimer reported below) and the effect of more distant species is more limited, especially because it could be “washed out” by thermal fluctuations of the duplex (not considered in our treatment).

Moreover, our predictions are solid not only with respect to the detail of the model adopted (inclusion of Na^+^ counterions, of explicit solvent molecules etc.) but also on the functional and the basis set used. Interestingly, in some cases there is a dependence on the location of the Ade^•+^ cation within the sequence. The spectra obtained for the external/Solvent exposed position was found to be in closest agreement (Figure S14) with the experimental one, which may reflect the slight opening of the base in the presence of the complex. Notably, our calculations predict a preference for locating the hole on the 5-′end. The effect of the nature of the stacked base (adenine vs thymine) is rather small, below the expected accuracy of our method. No significant delocalization effects of the hole on two stacked bases are found^63^ and the hole has a clear-cut preference for adenine bases, indicating that the oxidation of T is not a relevant process. Finally, as described in the SI, the computed difference spectra for a simple AT dimer in a WC hydrogen bonded arrangement are similar to those we have just discussed (see Figure S15), further supporting our assignment.

Considering the computational results, we therefore propose that the TRIR spectra can be explained by the processes described by eqs 5–7 below. In this sequence the intercalating dppz LC excited state is expected to oxidize adenine forming the **dppz**^**–•**^/(**A**^**+•**^**T**) contact radical ion pair (eq 5). The formation of [^4^Cr(TMP)_2_(^3^dppz)]^3+^ could occur either as a consequence of the back electron transfer (eq 6a) or by intersystem crossing from the [^4^Cr(TMP)_2_(^1^dppz)]^3+^/(AT) (eq 6b). This would be expected to be sensitive to the DNA binding site. At longer times (e.g., at 10 ns) the TRIR spectra show that the ^2^MC state has been formed.
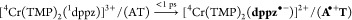
5
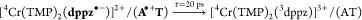
6a
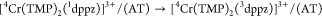
6b
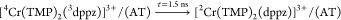
7

### ps-Time-Resolved Infrared (TRIR) in Mixed Sequence DNAs Sequences

**Figure 9:**
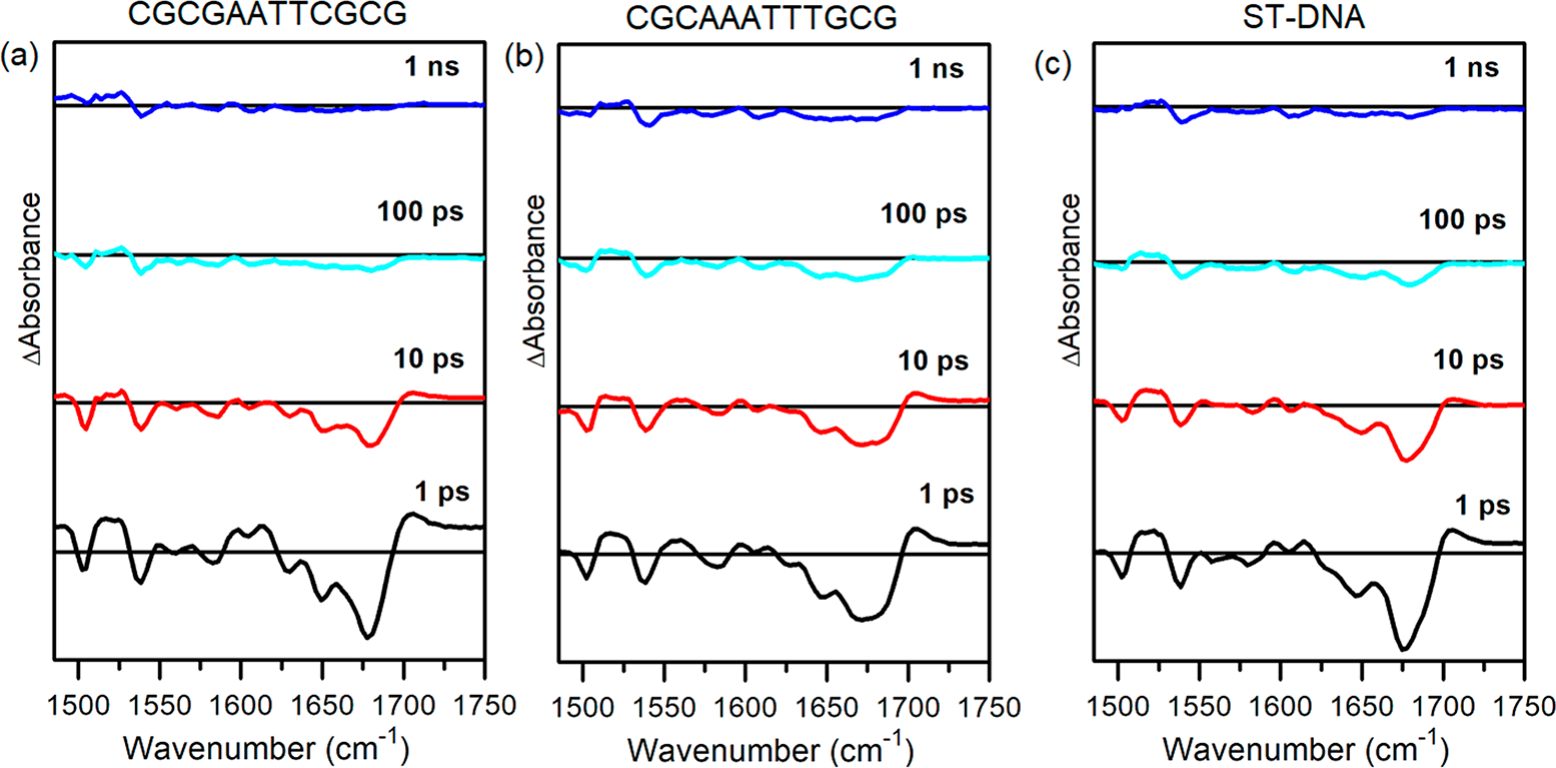
ps-ns-TRIR spectra (373 nm, 5 kHz, 1 μJ) of 1 mM of Λ-[Cr(TMP)_2_dppz]^3+^ in the presence of 25 mM nucleotide of (a) d(CGCGAATTCGCG)_2_, (b) d(CGCAAATTTGCG)_2_, and (c) ST-DNA DNA in 50 mM of sodium phosphate buffer in D_2_O.

Given the very different behavior observed for **1** in the presence of GC and AT DNA we next considered the behavior of **1** in the presence of the mixed sequence ODNs d(CGCGAATTCGCG)_2_ and d(CGCAAATTTGCG)_2_, which contain both GC- and AT-rich regions. These can then be compared to natural (ST-DNA) DNA which has 42% GC/58% AT. The TRIR data are presented in Figure 9 and Figure S17.

The spectrum recorded after 2 ps for **Λ-1** bound to d(CGCGAATTCGCG)_2_ is comparable to that recorded for d(GCGCGCGCGC)_2_ with strong nucleobase bleaches at 1649 and 1678 cm^–1^, slightly shifted compared to the homo-GC system (see Figure 9). Bleach bands of the complex are also seen at 1505 and 1538 cm^–1^. These observations suggest that photoexcitation predominantly affects G-C base pairs with less impact on the AT base pairs. In contrast to d(GC)_5_, a strong transient band at 1706 cm^–1^ is observed. This could be attributable to either the G^•+^C or A^•+^T species; however, no strong AT bleaching is observed, so we assign this to the guanine species. With **Λ-1** bound to d(CGCAAATTTGCG)_2_ the bleach pattern of the nucleobases is again dominated by those of G and C. However, there is an additional bleach contribution at 1690 cm^–1^, indicating an increase in the perturbation of AT base pairs. The absorption band 1705 cm^–1^ is even better defined than that observed for d(CGCGAATTCGCG)_2_. For ST-DNA the spectrum shows a clear absorption at high wavenumbers and some evidence of thymine bleaching, somewhat greater than found for d(CGCGAATTCGCG)_2_ but much less than for the complex bound to d(CGCAAATTTGCG)_2_ (see Figure 9).

**Figure 10:**
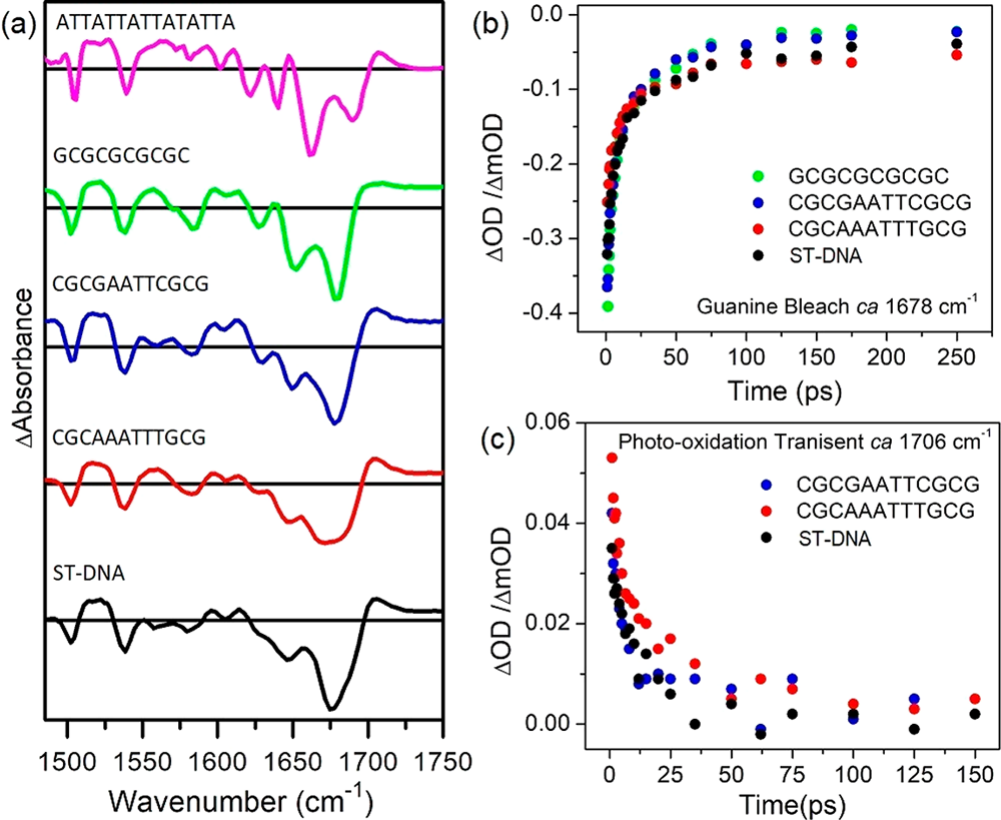
Comparative TRIR spectra of 1 mM of Λ-[Cr(TMP)_2_dppz]^3+^ in the presence of DNA systems at 1 ps (d(ATTATTATTATATTA)/d(TAATATAATAATAAT) at 2 ps) in 50 mM of sodium phosphate buffer in D_2_O. Kinetics showing recovery of (b) the guanine carbonyl bleach and (c) photo-oxidation transient.

Remarkably the spectra for these mixed sequence systems do not evolve substantially while they decay (see Figure 10a). Some residual bleaching of the nucleobase signals (1600 and 1700 cm^–1^) observed after the decay of the cation transient at >1700 cm^–1^ is attributed to perturbation by the presence of the excited state. They all show similar kinetics, which for the nucleobase recovery can be analyzed as a biexponential (Figure 10b–c). For d(CGCGAATTCGCG)_2_ and d(CGCAAATTTGCG)_2_ rapid nucleobase recovery with similar biphasic kinetics to those observed for the bleach bands of the complex so that the signals have substantially disappeared within 1 ns (Table 1, Figures S17–S18). Faster recovery of the cation transient at 1706 cm^–1^ is observed for these systems. These values are close to those found for the bleach recovery observed for d(GCGCGCGCGC)_2_, although in that case the process was attributed to back electron transfer of contact ion pair formed from the reduced metal complex and the guanine radical cation.

**Table 1:** Kinetic Analysis of DNA Systems

	**Λ-1**		**Δ-1**	
system	carbonyl bleach^a^ (ps)	transient *ca.* 1700^a^	carbonyl bleach^a^ (ps)	transient *ca.* 1700^a^
d(GCGCGCGCGC)_2_	3.7 ± 0.1 (63%); 42 ± 2 (37%)	6.0 ± 1.0^b^	5.2 ± 0.3 (80%); 52 ± 10(20%)	7.2 ± 2
d(CGCGAATTCGCG)_2_	4 ± 1 (65%); 40 ± 4 (35%)	4.4 ± 0.6 ps^b^	6.8 ± 1.0 (74%); 55 ± 10(26%)	7.0 ± 0.8
d(CGCAAATTTGCG)_2_	4 ± 1 (63%); 49 ± 4 (37%)	2.0 ± 0.5 (63%); 28 ± 4 (37%)	3.9 ± 1.0 (43%); 49 ± 12(57%)	3.6 ± 1.5(43%); 39 ± 4(57%)
ST-DNA	5 ± 0.5 (61%); 72 ± 7 (39%)	10 ± 1^b^	8.6 ± 0.8 (67%); 101 ± 15 (33%)	2.0 ± 1.0 (60%); 31 ± 5 (40%)

a Baseline referencing applied (1737 cm^–1^).

b Best fit to a monoexponential.

### Delta Enantiomers and DNA Systems

The major features in the transient spectra of the delta enantiomer **Δ-1** bound to the various ODN systems are broadly similar to those found with its lambda counterpart (Figure S17). With d(GCGCGCGCGC)_2_ the absorption band at 1703 cm^–1^ is better defined and the valley between the G and C carbonyl stretches is shallower (Figure S19). This latter feature is accentuated for d(CGCGAATTCGCG)_2_, where the C band almost becomes a shoulder on that of the G. For d(CGCAAATTTGCG)_2_ the spectrum shows features associated with T, as was found with the lambda enantiomer. With natural DNA the bleach profile for the delta enantiomer more closely resembles d(CGCAAATTTGCG)_2_. In general, the kinetics are longer lived in the case of the delta enantiomer (Figure S20) which likely reflects the differences in binding.

## Discussion

These experiments reveal that unlike photo-oxidizing dppz complexes of rhenium^7,9^ and ruthenium,^10^ [Cr(TMP)_2_dppz]^3+^ (**1**) is able to oxidize adenine. The effect is demonstrated by the partial quenching observed in the titration observed with the ODN containing only AT base pairs (Figure 2). The origin of this quenching is due to access to relatively long-lived dppz-localized upper excited states upon irradiation at 373 or 400 nm, as revealed by TA and TRIR experiments. The reduced quenching observed for the AT system is attributed to the fact that the electron transfer from the dppz LC excited state is only partially competitive with the nonradiative processes leading to the emissive MC state. The time-resolved experiments indicate the formation of at least two dppz-localized excited states which lead finally to the ^2^MC state. Significantly, these various excited states appear to have characteristic TRIR spectra. We assign the first of these to [^4^Cr(TMP)_2_(^1^dppz)]^3+^ and with the aid of the TRIR spectrum supported by DFT calculations assign the next state to the [^4^Cr(TMP)_2_(^3^dppz)]^3+^ species formed by intersystem crossing. It should also be noted that LMCT states are expected to lie energetically between the LC states and the lowest lying ^2^MC state. Although we have no definitive evidence for such LMCT states, it is possible that the species decaying with a lifetime of 4 ns is such an excited state. The identification of the LC upper excited states is highly significant, as until now discussion of the dynamics of Chromium polypyridyl complexes in terms of photocatalysis^26,64^ and DNA photodamage^32,36,51^ has been dominated by the role of the ^2^MC state and the ability to tune its energy through ligand substitution.^65^

**Figure 11:**
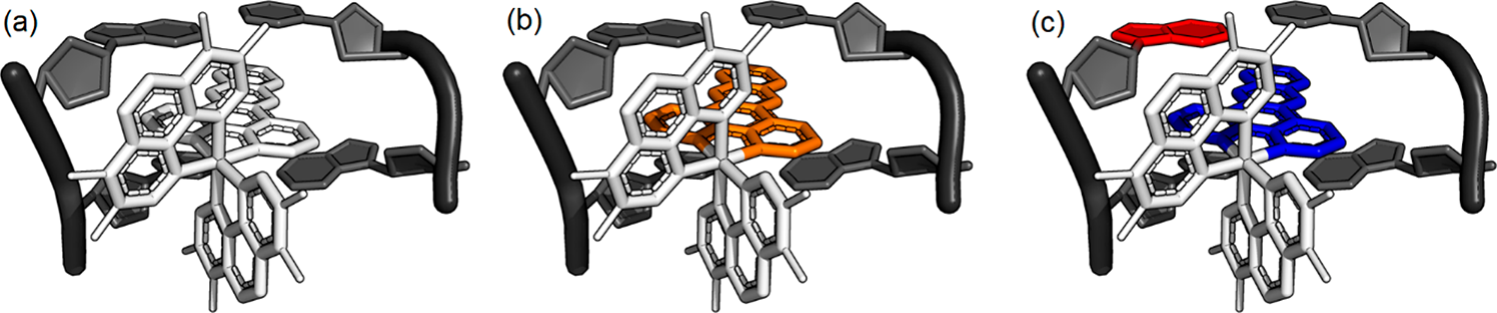
Schematic example of expected changes in electron density for Λ-1 intercalated into a (a) base-pair step upon (b) formation of the LC excited state and (c) subsequent electron transfer. Orange = LC excited state, red = oxidized purine, blue = reduced dppz ligand.

The strong binding affinity of **1** for DNA places the intercalating dppz ligand in direct contact with the base pair (see Figure 11a). Critically, this now allows the relatively short-lived LC dppz excited state ([^4^Cr(TMP)_2_(^1^dppz)]^3+^) to participate in electron transfer processes (see Figure 11b–c). The absorption spectrum indicates that this LC state lies *ca*. 120 kJ mol^–1^ higher in energy than the ^2^MC state, for which the excited state reduction potential, *E*^0^(*Cr^3+^/Cr^2+^) = 1.39 V, has been reported. Thus, a value of *E*^0^(*Cr^3+^/Cr^2+^) ca. 2.6 V may be calculated for the LC species.^32^ When bound to guanine-containing DNA, we propose that the initially formed excited state, i.e. [^4^Cr(TMP)_2_(^1^dppz)]^3+^, undergoes subpicosecond electron transfer with the nucleobase to form [^4^Cr(TMP)_2_(dppz^•–^)]^2+^/(G^•+^C). This species has a distinct TRIR spectrum in which the metal complex is associated with the GC base pair, as evidenced by the strong nucleobase bleaching (Figure 6). These results are similar to what we previously reported for *rac*-[Cr(phen)_2_(dppz)]^3+^, which also undergoes rapid quenching.^35^ In the case of DNA containing only AT base pairs, a striking observation is that excitation of **1** produces a product having a characteristic absorption band at ca. 1708 cm^–1^ and a lifetime of 27 ps (Figure 7). DFT calculations indicate that such an absorption band is expected for a thymine HB to an adenine radical cation (**A**^**•+**^**T**) in a WC base pair (Figure 8).

In mixed DNA sequences the TRIR spectra of both **Λ-1** and **Δ-1** indicate the formation of a short-lived reversible electron transfer photoproduct having a strong transient band observed at ca. 1706 cm^–1^ (Figure 9 and Figure S17). Critically, the bleaches predominantly associated with the GC base pairs are taken to report on the site of photo-oxidation. The recovery to the ground state occurs rapidly (<100 ps) with similar biexponential kinetics, which may arise due to the complex being in different binding sites or in different orientations (Figure 10).^9,66^ This is interesting as previous equilibrium dialysis of Λ-**1** with DNA demonstrated a marked preference for alternating AT over alternating GC base sequences.^32^ Furthermore, as ST-DNA is a heterogeneous system with up to 10 distinct base pair steps and 42% GC composition, some photo-oxidation would be expected to occur at sites containing AT and GC steps. The observation in ST-DNA of GC as the dominant site of photo-oxidation may be explained by the fact that even if the complex intercalates at an AT/AT or a TA/TA base pair step, charge transfer by hole migration to guanine would be a favored process.^67−69^ Importantly, by 2 ps in natural DNA oxidation is localized predominantly at GC sites indicating that any hole transfer is very rapid and is found to occur for both enantiomers.

Our results demonstrate the photo-oxidizing behavior of **1** to be strikingly different from that of other metal complexes which are known to cause one-electron-oxidation of DNA and where transient spectroscopic experiments have been carried out. Notably, the electron transfer dynamics of **1** are extremely fast, occurring within 100 ps in mixed DNA. This contrasts with the behavior of the extensively studied [Ru(TAP)_2_(dppz)]^2+^ complex where electron transfer from guanine to generate the G^•+^ radical cation species proceeds with a forward rate of about 1/500 ps and back reaction with lifetimes between 5 and 20 ns depending on the sequence.^10,40,44,70^ The slower dynamics for [Ru(TAP)_2_(dppz)]^2+^ can be explained by electron transfer occurring from guanine to the Ru(III) center of the MLCT state which is more removed from the GC base pair than is the case for **1** and by the back reaction between the oxidized guanine and the reduced ancillary TAP ligand, which are relatively far apart and orthogonal to each other.

In a detailed study of intercalating rhenium carbonyl compounds, Barton, Vlcek and co-workers investigated a tethered [Re(CO)_3_(dppz)py]^+^ derivative bound to oligonucleotides containing either only GC or only AT base pairs as well as mixed sequences.^9^ They concluded that oxidation of guanine by the excited state could only occur if the metal complex was intercalated at a G-C basepair. Indirect evidence for very rapid electron transfer to guanine from LC and MLCT states was reported. However, significantly they showed that charge migration through the DNA basepair stacks could occur. George, Kelly and co-workers similarly reported that with [Re(CO)_3_(F_2_dppz)py]^+^ 66% underwent subpicosecond electron transfer and 34% with a lifetime of 34 ps when the complex was bound to double-stranded polydGC.^7^ As with **1** these studies both propose a role for LC states oxidizing guanine on a subpicosecond time scale, but unlike in our case being unable to oxidize adenine. This is presumably because the redox potential of the excited state of the rhenium complexes is insufficient to do so. With **1**, as was pointed out earlier, the LC is predicted to be a very strong oxidizing agent (estimated (*E*^0^(*Cr^3+^/Cr^2+^) ca. 2.6 V).

Evidence of adenine photo-oxidation by transition metal complexes is extremely rare. An exceptional study reported the photo-oxidation of adenine by the MLCT state of the highly oxidizing Ru(tap)(hat)_2_^2+^ and Ru(hat)_3_^2+^ (HAT = 1,4,5,8,9,12-hexaazatriphenylene) when bound to poly(dA-dT) (albeit bound more weakly than dppz complexes), observed indirectly by quenching of emission of these complexes.^71^ Yet, until now the direct observation of sensitized adenine photooxidation to generate the adenine radical cation has not been observed. In this study our combined TRIR and DFT characterization has allowed the identification of the elusive adenine radical cation (**A**^**•+**^**T**), which is formed due to the proximity of the dppz LC upper excited state of **1** when intercalated in DNA.

## Conclusion

This study reveals the key role of a second and more potent LC oxidizing state of the chromium polypyridyl complex **1** in the mechanism of DNA photodamage. While the ^2^MC state is expected to participate in photo-oxidation observed in the case of diffusional quenching, when **1** is intercalated the preorganization of the dppz ligand adjacent to Watson Crick base pairs facilitates photo-oxidation to be dominated by the LC state, circumventing the ^2^MC state. TRIR and DFT reveal that, when **1** is bound exclusively to AT ODNs, photoexcitation generates the radical adenine cation (**A**^**•+**^**T**). Furthermore, in mixed DNA systems binding at an AT site likely leads to hole migration to form the guanine radical cation. In contrast to other systems, this enhanced photoactivity, irrespective of binding site, contributes to the significant formation of the guanine radical cation in natural DNA. These observations have important implications for the application of chromium polypyridyl complexes as both photocatalysts and nucleic acid targeting therapeutics. Our increased understanding of the role of ligands in transition-metal-mediated catalysis^72^ has placed focus on chromium’s rich redox chemistry.^73^ Preorganization of the substrate could be used to enhance the photocatalytic activity of these complexes in radical cation mediated reactions.^26^ Ruthenium polypyridyl complexes are actively pursued as antimicrobial agents;^74,75^ however, the combined photoactivity and affinity of **1** toward AT-rich DNA opens the possibility of improved activity through targeting AT-rich repeated sequences of bacterial replicons.^76^ Additionally, as an earth-abundant element, chromium complexes are economical and sustainable constituents for these applications. It is clear that the photochemistry of chromium polypyridyl complexes allows them to boldly go where no complexes have gone before.

## Experimental Section

5-Guanosine monophosphate (GMP) and deoxy adenosine monophosphate (dAMP) were supplied by Sigma-Aldrich. 5′-GCGCGCGCGC-3′ (84 400 M^–1^ cm^–1^), 5′-CGCGAATTCGCG-3′ (101 700 M^–1^ cm^–1^), 5′-CGCAAATTTGCG-3′ (112 500 M^–1^ cm^–1^), 5′-ATTATTATTATATTA-3′ (158 300 M^–1^ cm^–1^), and 5′-(TAATATAATAATAAT)-3′ (167 200 M^–1^ cm^–1^) were synthesized by Eurogentec (Liege, Belgium) and obtained in HPLC purified form. Oligodeoxynucleotide concentrations were determined spectrophotometrically using the single-strand extinction coefficients at 260 nm shown in parentheses and made up in D_2_O in the presence of 50 mM potassium phosphate buffer, pH 7. Solution samples for transient spectroscopy measurements were prepared by dropping a known volume of solution (25–35 μL) between two CaF_2_ (25 mm diameter) windows (Crystran Ltd., UK), separated by a Teflon spacer of 50 μm path length, in a demountable solution IR cell (Harrick Scientific Products Inc., New York).

[Cr(TMP)_2_(dppz)]^2+^ (**1**) was synthesized using previously reported methods.^32^ The chiral resolution of **1** was achieved by cation exchange chromatography following a method described by Vasudevan et al.^33^ First, 40 mg of **1** as PF_6_^–^ salt were exchanged to the chloride form by stirring a methanolic solution over Dowex 1X8-200 for 2 h, followed by evaporation to dryness. The racemic mixture of the complex was dissolved in water (1 mL) and loaded onto a sephadex C-25 stationary phase with a (−)-*O,O*′-dibenzoyl-l-tartrate chiral eluent. The column was run at a flow rate of 2 mL/min. The resolved bands were collected into several centrifuge tubes (12 fractions, 5 mL each). The two separate fractions were immediately converted from the water-soluble tartrate salts to the PF_6_^–^ salts by addition of ammonium hexafluorophosphate (NH_4_PF_6_). This was done to prevent racemization, which has been observed for solutions of the tartrate salts. The yellow precipitate was collected by centrifugation (3000 rpm; 3 min) and washed with water (0.5 mL). Then, lambda and delta enantiomers were dissolved in a minimum volume of acetone and stirred at room temperature using a chloride anion exchange resin (Dowex 1X8-200) in methanol for 2 h. After that period, the solutions were filtered and evaporated to dryness. The concentration of the complex was determined using the molar extinction coefficient 16 600 M^–1^ cm^–1^ at 360 nm.^32^

### Instrumental Methods

UV/vis absorption spectra were recorded on a Varian Cary 200 spectrophotometer. Steady-state luminescence spectra were recorded on a Varian Cary Eclipse. Circular dichroism measurements were recorded on a JASCO J180. Infrared spectra were recorded on a Nicolet Avatar FT-IR spectrometer or Varian 3100 FT-IR spectrometer fitted with a Universal ATR Sampling Accessory for solid samples or N_2_ flushed liquid samples between CaF_2_ plates with an optical path length of 100 or 150 μm and at a resolution of 4 cm^–1^ and 256 scans.

### Computational Methods

#### Metal Complex

All quantum chemical calculations on the metal complexes were performed using Gaussian 16 (Revision B.01) as implemented at the Irish Centre for High-End Computing.^77^ Molecular structures were optimized to tight convergence criteria. The molecular structure and density maps were visualized using GaussView 6,^78^ while fragment contributions to orbitals were extracted using AOMix.^79,80^ The unrestricted B3LYP hybrid Density Functional Theory (DFT) was used,^81,82^ coupled to the double-ζ quality LanL2DZ basis set.^83^ A similar approach proved successful in characterizing the nature of excited states and the normal modes of related ruthenium systems.^41,55^ At each optimization, the Hessian matrix was found to contain no negative elements, which confirms that the structures sit in a minimum on the potential energy hypersurface. Corrections for solvent dielectric (water) were applied in these calculations using the Polarizable Continuum Model (PCM) and the integral equation formalism variant (IEFPCM).^84^

#### Ade^•+^ in AT Steps Containing Adenine Cation

Several models have been used for describing duplexes containing the AT step (SI). Their “neutral” and cationic form were optimized, in which one of the bases was in its cationic form. All the calculations have been performed at the DFT level, using the M052X^85^ functional combined with the 6-31G(d) basis set. Solvent effects were also considered using PCM^86,87^ and in some cases by inclusion of specific water molecules (SI). Harmonic spectra were built by inclusion of a broadening at each transition with a Gaussian of h.w.h.m = 10 cm^–1^ and uniformly scaled by a factor of 0.955. This procedure has been satisfactorily applied to model the spectral properties of oligonucleotides (including their IR spectra)^58,88,89^ and the behavior of adenine cation within DNA, providing IR spectra in good agreement with their experimental counterparts.^90−92^ Test calculations were also performed on a simpler model, i.e. 9methyl-A:1methyl-T dimer in a WC arrangement (see SI), by varying the density functional and/or the basis set. The computed difference spectra are fully consistent with those obtained on larger systems with M052X/6-31G(d). Our conclusions are thus solid with respect to the adopted computational model, functional and basis set.

### TRIR Measurements Using LIFEtime Apparatus

TRIR measurements were performed using the LIFEtime apparatus at the Central Laser Facility.^93,94^ Briefly, a dual Pharos regenerative amplifier system (Light Conversion) was used to generate 1030 nm synchronized probe (100 kHz, 50 μJ, 180 fs) and pump (0.01–50 kHz, 150 mJ, 260 fs) pulses. The probe laser was used to drive two OPAs (Orpheus-ONE, Light Conversion), which generated tunable mid-IR probe light via successive steps of optical parametric generation in BBO, KTA and difference frequency generation in GaSe. The two independently tunable mid-IR probe beams were focused through the sample with <70 μm spot sizes (fwhm) and dispersed onto two separate 128 pixel MCT array detectors (IR Associates). The second harmonic of the pump laser (515 nm) was used to pump a BBO-based OPA (Orpheus-HP, Light Conversion), generating 373–400 nm pump light from the second harmonic of the signal. The 373–400 nm pump light was delivered to the sample via a 1.2 m length two-pass delay stage (Newport) and focused through the sample with a spot size of 150 μm fwhm (pump–probe polarization “pp” was set at magic angle). Pump–probe delays were set using a combination of the delay stage (0–16 ns) and seeding of the two regenerative amplifiers with different seed pulses from their common oscillator (giving delays in 12 ns out to 10 ms). In the present experiments, the samples were observed to recover on a μs time scale, and so 50 kHz pumping in combination with rastering of the sample was used. The 400 nm pump energy was 200 nJ.

### ps-TA (373 nm) and ns-TA (355 nm) Measurements

The TA experiments were performed on ULTRA apparatus at the Central Laser Facility.^94^ For the ps-TA measurements, part of the titanium sapphire laser output beam was used to generate a white light continuum (WLC) in a CaF_2_ plate. The crystal plate was continuously rastered to avoid color center formation and to improve stability in the probe. The WLC was dispersed through the grating monochromator and detected using a linear silicon array (Quantum Detectors). In front of the monochromator, a long-pass filter was placed in order to remove scatter from the excitation beam. The 373 nm pump beam (pulse length ca. 50 fs) was provided by the OPA (TOPAS, Light Conversion) pumped with the fundamental beam of the 10 kHz titanium sapphire laser. For ns-TA, the same setup was used; however, the pump beam was provided by the Q-switched ns laser (InnoLas, pulse length 1 ns) electronically synchronized with the main 10 kHz titanium sapphire amplifier. The third harmonic output (355 nm) of that ns laser was used to excite the samples. The polarization of the pump pulses at the sample was at the magic angle relative to the probe, with an energy of 1 μJ and a spot size of ca. 100–150 μm. The spectra were calibrated using five band-pass filters.

## Supporting Information Available

Methods for binding constant determination, additional computational methods, DNA spectroscopic titrations, additional ps/ns-TA and ps/ns-TRIR spectra, kinetic fits, calculated TRIR spectra, spectral assignments, and binding constants (PDF)
