# Supplementary material for: Adenine Radical Cation Formation by a Ligand-Centered Excited State of an Intercalated Chromium Polypyridyl Complex Leads to Enhanced DNA Photo-oxidation
Source: J Am Chem Soc. 2021 Aug 31;143(36):14766–79. doi: 10.1021/jacs.1c06658 (PMC8447253; doi:10.1021/jacs.1c06658)
Supplement: Supplementary file 1 — ja1c06658_si_001.pdf [file ja1c06658_si_001.pdf]

Supporting Information

Adenine Radical Cation Formation by a Ligand-Centered Excited State of an Intercalated Chromium Polypyridyl Complex Leads to Enhanced DNA Photo-oxidation

Frederico A. Baptista,[1] Dorottya Krizsan,[1] Mark Stitch,[1] Igor V. Sazanovich,[2] Ian P. Clark,[2] Michael Towrie,[2] Conor Long,[3] Lara Martinez-Fernandez,[4] Roberto Improta,[5] Noel A. P. Kane-Maguire,[6] John M. Kelly[7], Susan J. Quinn[1]

e-mail: jmkelly@tcd.ie susan.quinn@ucd.ie

[1]*School of Chemistry, University College Dublin, Dublin 4, Ireland.*

[2]*Central Laser Facility, Research Complex at Harwell, STFC Rutherford Appleton Laboratory, Oxfordshire, OX11 0QX, UK*

[3]*The School of Chemical Sciences, Dublin City University, Dublin 9, Ireland.*

[4]Departamento de Química, Facultad de Ciencias and Institute for Advanced Research in Chemistry(IADCHEM) Universidad Autónoma de Madrid, Campus de Excelencia UAM-CSIC, Cantoblanco, 28049 Madrid, Spain

[5]*Consiglio Nazionale delle Ricerche, Istituto di Biostrutture e Bioimmagini, 80136 Naples, Italy*

[6]*Department of Chemistry, Furman University, 3300 Poinsett Highway, Greenville, SC 29613-1120, USA*

[7]*School of Chemistry, Trinity College Dublin, The University of Dublin, Dublin 2, Ireland.*

Section S1 Methods

S1 DNA Binding Constant Calculations

The method described by Bard *et al.*[1] was used to fit the emission data using equation (**1**) shown below. The binding constant of the enantiomers binding to DNA structures was determined by fitting equation (**1**) to a non-linear plot of (I_a – I_f)/(I_b – I_f) vs [DNA], for molar emission (with [DNA] concentration in nucleotides, fitting using Origin 8.5 software).

$$\frac{(I_a - I_f)}{(I_b - I_f)} = (b - (b^2 - 2k^2 C_t[DNA]/s)^{\frac{1}{2}})/2kC_t \qquad (\mathbf{1})$$

where $b = 1 + kC_t + k[DNA]/2s$

And; k is the equilibrium binding constant in M^{-1}, C_t is the total metal complex concentration (changes to this are only due to dilution by the addition of DNA), s is the binding site size and [DNA] is the concentration of DNA in nucleotides. I_f is the molar emission of free metal complex (I_f = initial E_m /Ct), the apparent molar emission (I_a = apparent E_m/C_t) and the molar emission of the metal complex in the fully bound form (I_b = Final E_m/C_t), E_m = emission

S2 Computational Methods

Metal Complexes

Quantum chemical calculations on metal complexes. All quantum chemical calculations on the metal complexes were performed using Gaussian 16 (Revision B.01) as implemented at the Irish Centre for High-End Computing.[2] Molecular structures were optimised to tight convergence criteria. The molecular structure and density maps were visualised using GaussView 6,[3] while fragment contributions to orbitals were extracted using AOMix.[4-5] The unrestricted B3LYP hybrid Density Functional Theory (DFT) was used,[6-7] coupled to the double zeta quality LanL2DZ basis set.[8] At each optimisation the Hessian matrix was found to contain no negative elements, which confirms that the structures sit in a minimum on the potential energy hypersurface. Corrections for solvent dielectric (water) were applied in these calculations using the Polarizable Continuum Model (PCM) and the integral equation formalism variant (IEFPCM).[9] Normal mode energies were expressed as Gaussian functions (half-width at half maximum 4 cm^{-1}) and uniformly scaled by 0.973. This model chemistry has been used successfully to simulate the IR spectra of both ground state and excited states of other metal complexes.[10-11] All UV/visible spectra were simulated using TD-DFT calculations at the same model chemistry as used for the ground state.

$Ade^{\bullet+}$ in AT steps containing Adenine cation.

We have optimized several models of duplexes containing the AT step. In detail: (i) the AT Watson-crick base-pair (WC); (ii) the dinucleotide dApT (AT), also including two Na^+ counterions (hereafter ATNa). In this case we have also studied a model explicitly including two D_2O molecules (hereafter denoted as AT·2 D_2O); and (iii) the trinucleotides TpdApT (hereafter TAT), including four Na^+ counterions (hereafter TATNa, see below) and dApTpT (hereafter ATT).

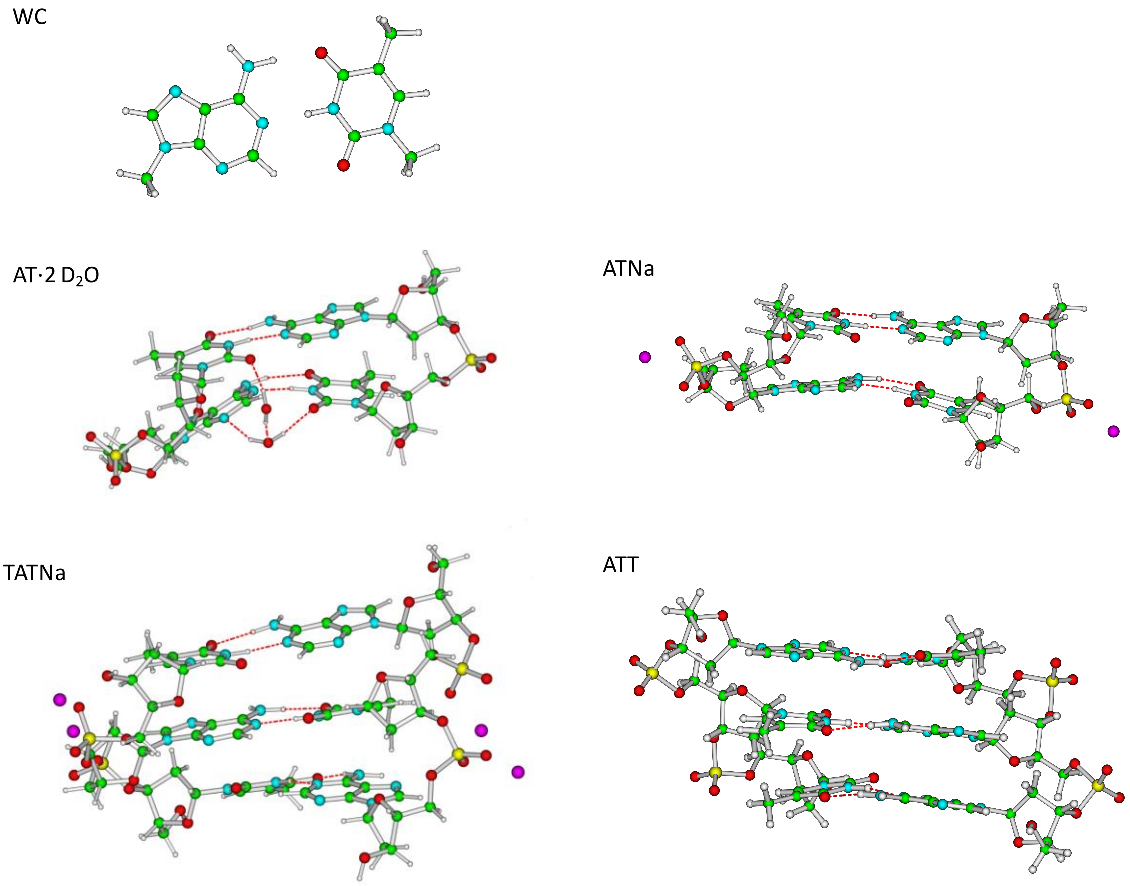

Scheme S1: Schematic depiction of the structures of the computational models for AT DNA.

Solvent. All the calculations have been performed including solvent (water) effect by the Polarizable Continuum Model (PCM). As anticipated below, in some cases two explicit water molecules were also considered in addition to PCM.

IR and difference IR Spectra. The IR spectrum for the neutral and cationic minima were computed at the harmonic level. To account for the lack of modelling of anharmonic effects and to facilitate comparison with experiments, all frequencies were uniformly scaled by 0.955 and convoluted with a Gaussian function of half width half maximum of 10 cm^{-1}. The labile protons were substituted with deuterons.

Note on Characterizing the spectral signatures of $Ade^{\bullet+}$ in ODN

In addition to the calculations on the trimer fragments several test calculations on the simple 9methylAdenine-1methylThymine WC dimer, in its neutral and cationic form were performed. These allow better decomposition of the different effects modulating the IR spectra and confirm the reliability of our conclusions. For the WC system we have computed the IR spectrum of the 'neutral' and the cationic species not only by using M052X/6-31G(d) calculations, but also the B3LYP-D3 (both with 6-31G(d) and 6-311+G(d,p) basis set) and CAM-B3LYP-D3 (with the 6-31G(d) basis set) functionals. As shown by the IR spectra reported below (see Figure S15) our conclusions are solid with respect to the adopted computational model, functional and basis set.

In the ATT system, three Ade bases are present. Two are exposed to the solvent, on the 5'-ter end of the two strands, whereas one is 'internal'. We did not succeed in finding a stable minimum for a cationic species when Ade^{+} was in this latter position, confirming the preference for locating the hole on the solvent exposed 5'-end. In one of the two strands we find two stacked Ade bases (TAA), whereas in the other Ade is stacked on T. We located the 'cationic' minimum for both species, i.e. $A^{+}AT$ and $A^{+}TT$. Their stability is very similar, $A^{+}AT$ being more stable by 0.03 eV, i.e. below the expected accuracy of our calculations. This result confirms that the hole is essentially localized on a single A base. The computed difference spectra for both species (see Figure S16) are qualitatively similar to those obtained with the other models of AT DNA.

Note: The position and the intensity of the different peaks slightly changes depending on the considered sequence and can be sensitive to HB with D_2O, especially the C2=O2 stretching, which is not involved in the WC pairing. As a consequence, considering that rather flexible AT ODN would explore different conformations, while our calculations are based on single minima, we can expect that the experimental spectrum would be less resolved than the computed one. Nonetheless, the comparison between the computed and experimental IR spectra is good, supporting the reliability of our computational approach.

Section S2 Figures

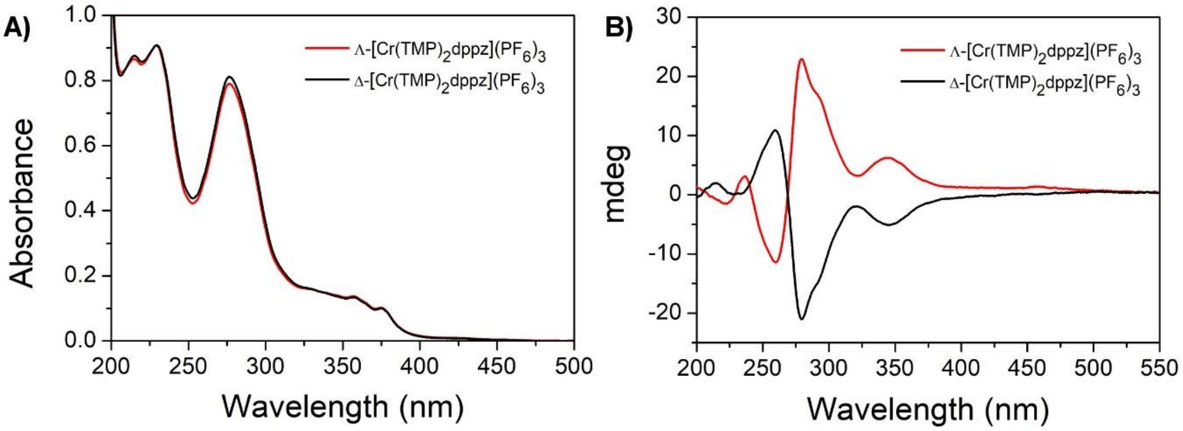

Figure S1 UV-Vis spectrum (A) and CD spectrum (B) of Δ–1 (7.95 μM) and Λ–1 (7.75 μM) enantiomers in acetonitrile. Cell pathlength = 1 cm.

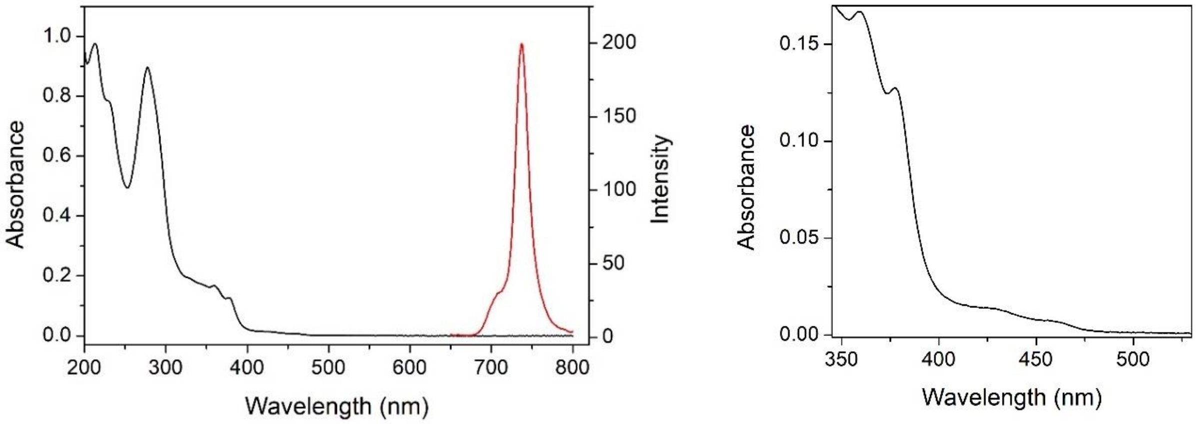

Figure S2. Left: UV-visible absorption and emission spectrum of 10 μM of **rac-[Cr(TMP)$_2$dppz]$^{3+}$** in presence of 50 mM potassium phosphate buffer. Excitation at 380 nm. Right: UV-visible spectrum of visible transitions.

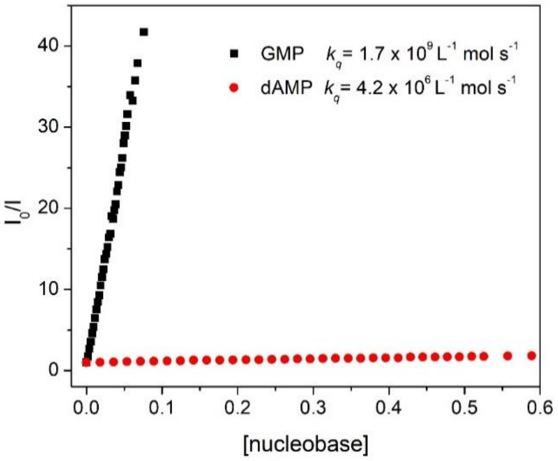

Figure S3: Stern-Volmer plots for the steady state emission quenching I_0/I of an air-saturated 13 $\times$ 10^{-6} M solution of **[Cr(TMP)$_2$(dppz)]$^{3+}$** in 50 mM phosphate buffer (pH 7.0) by GMP and dAMP steady-state emission

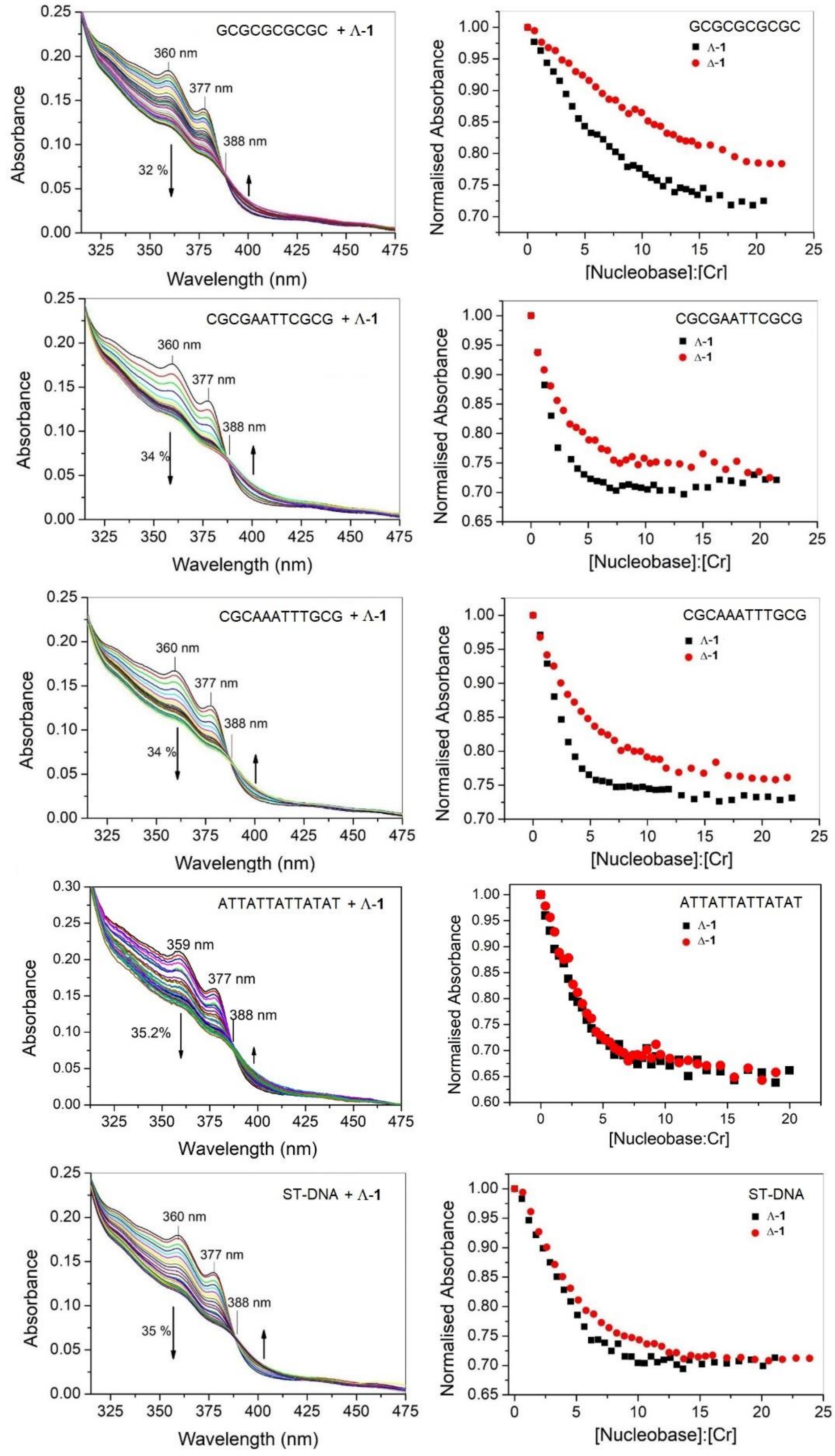

Figure S4. Left: UV-Vis spectrum of **Λ-[Cr(TMP)$_2$dppz]$^{3+}$** increasing concentrations of DNA systems, all in presence of 50 mM potassium phosphate buffer, pH 7. Right: Comparative changes at 360 nm for both enantiomers.

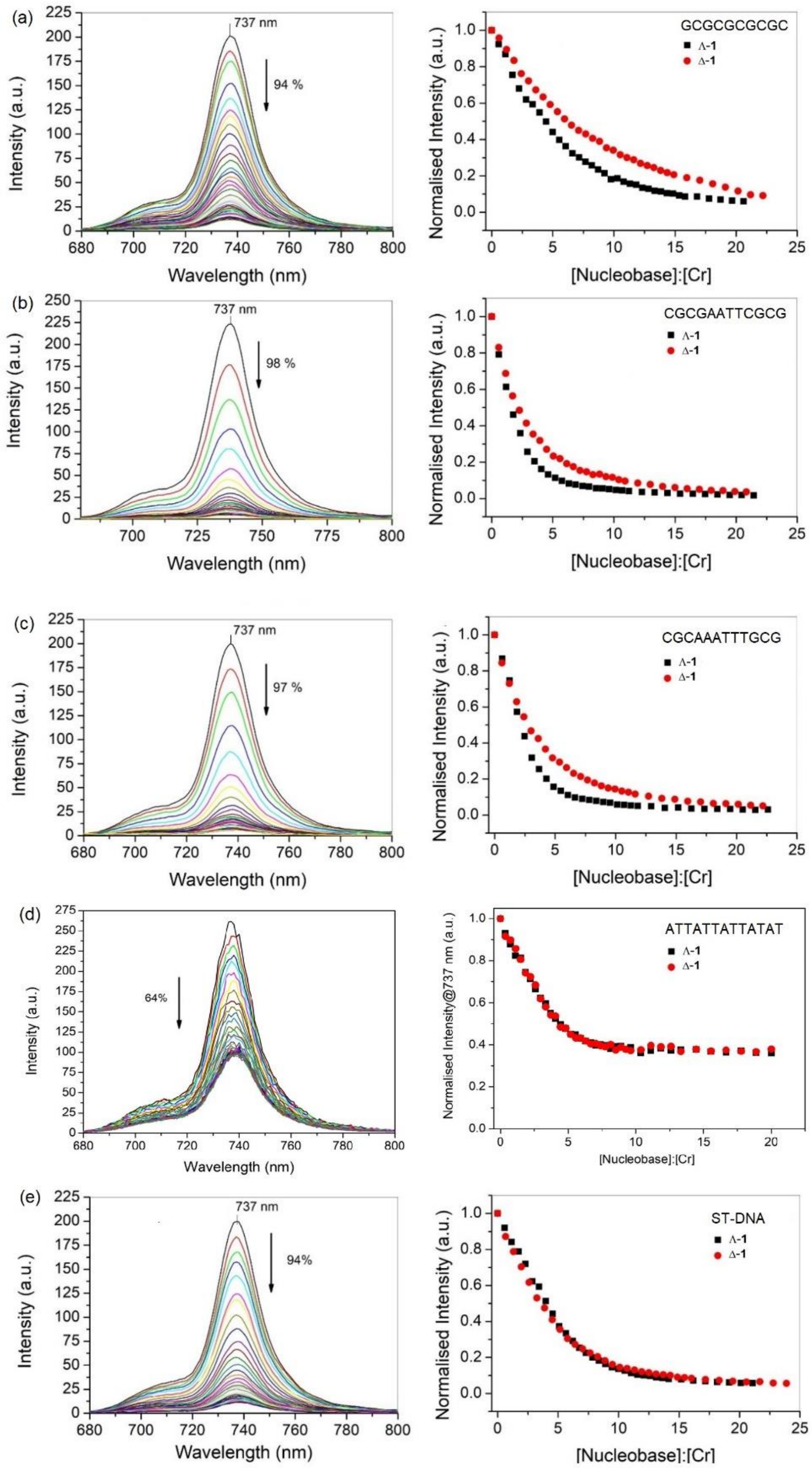

Figure S5. Emission spectrum of 10-11 μM **Λ-[Cr(TMP)$_2$dppz]$^{3+}$** in presence of 50 mM phosphate buffer and increasing concentrations of DNA (0-230 μM) upon excitation at 380 nm (left). Normalised titration intensity compared to the change observed for **Δ-[Cr(TMP)$_2$dppz]$^{3+}$** (right).

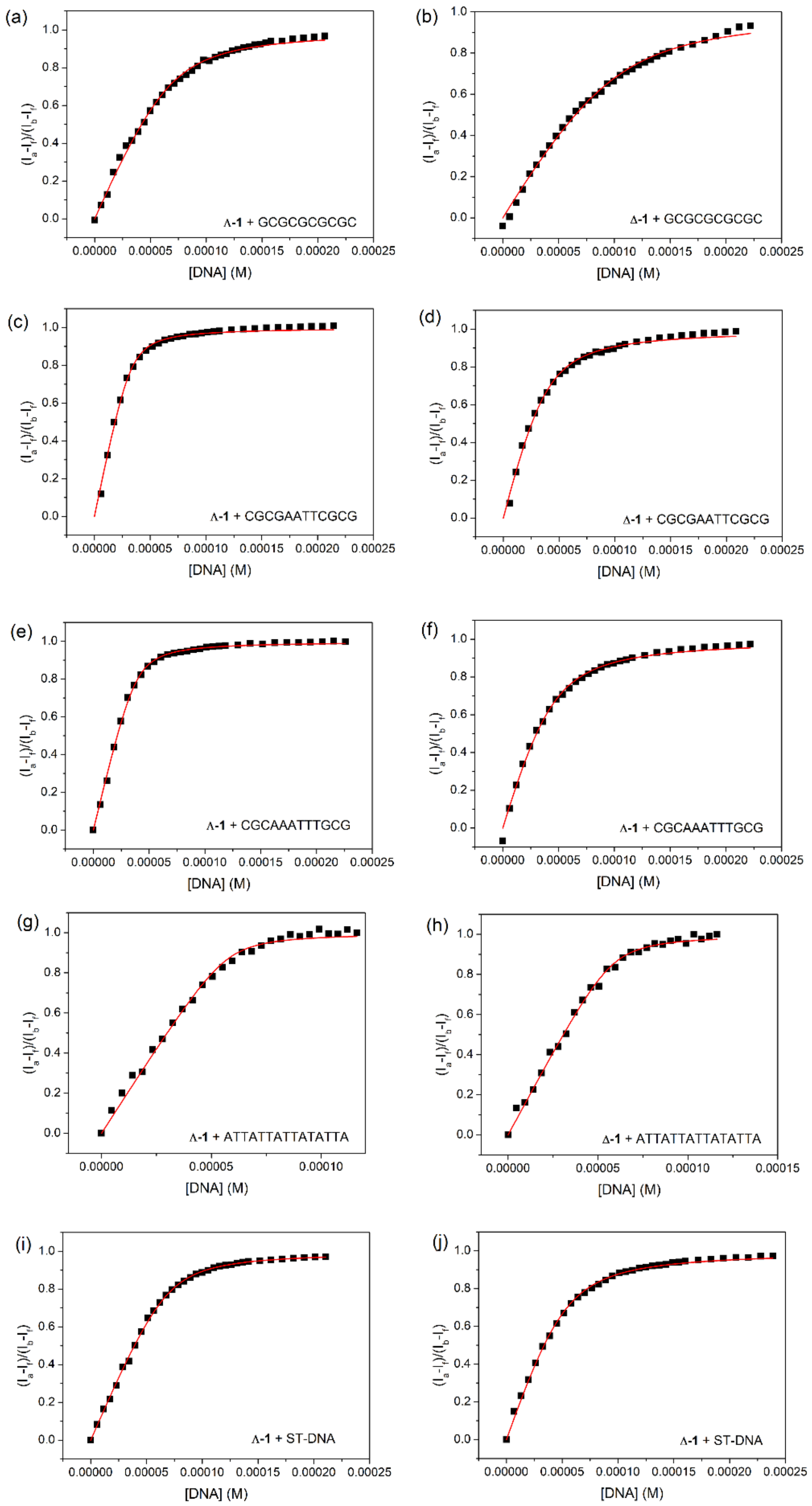

Figure S6. Determination of the binding constant for **Λ-[Cr(TMP)$_2$dppz]$^{3+}$** and **Δ-[Cr(TMP)$_2$dppz]$^{3+}$** (10-11 μM) in the presence of different DNA systems. Plots of (I_a-I_f)/(I_b-I_f) at 737 nm vs. [DNA] and the best fits of the data (---) using the method of Bard.[1] DNA concentrations expressed in nucleotides. All data in aerated 50 mM phosphate buffer pH 7.0.

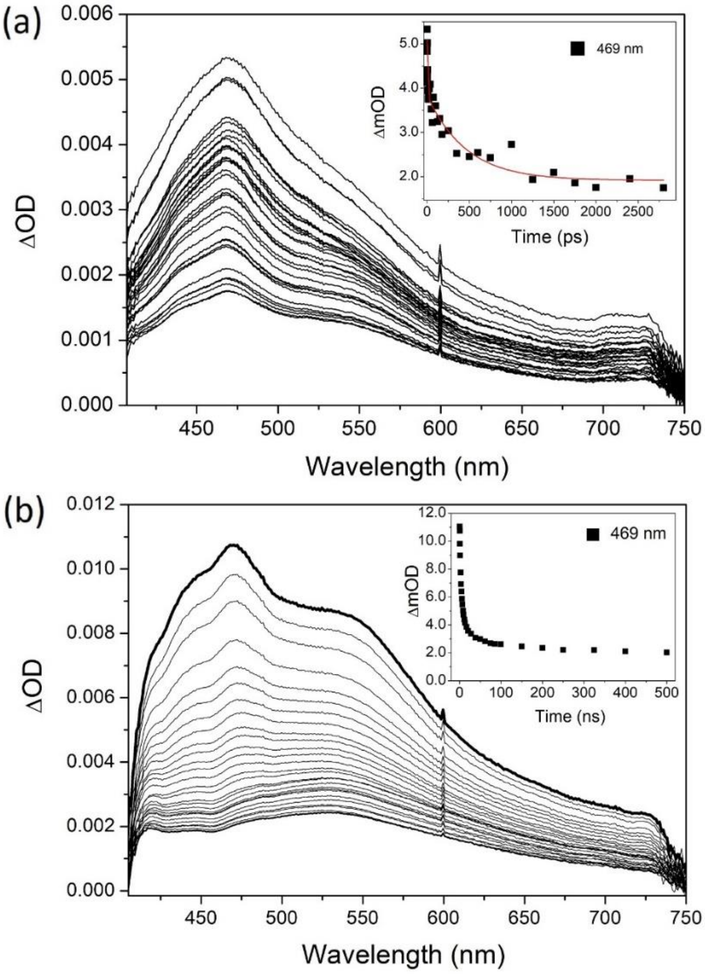

Figure S7. (a) Transient absorption spectra recorded from 1 ps to 2800 ps following 373 nm excitation of a D_2O solution of **[Cr(TMP)$_2$(dppz)]$^{3+}$** (1 mM). Inset shows the decrease of absorption monitored at 469 nm. (b) Transient absorption spectra recorded from 1 ns to 500 ns following 355 nm excitation of a D_2O solution of **[Cr(TMP)$_2$(dppz)]$^{3+}$** (2 mM). Inset shows the decrease of absorption monitored at 469 nm.

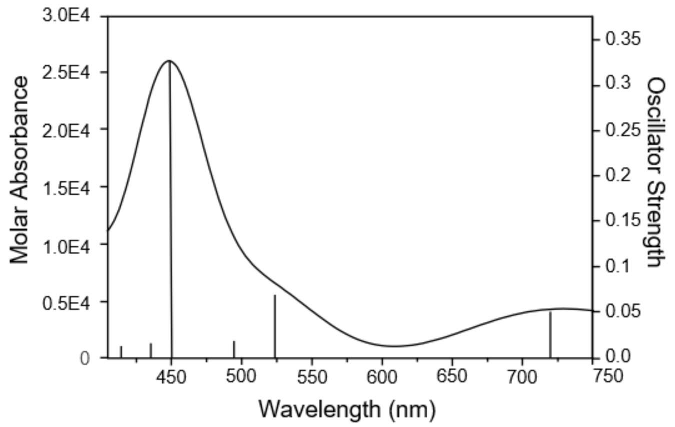

Figure S8: Simulated UV-visible absorption spectrum of $[^4Cr(TMP)_2{}^3dppz]^{3+*}$obtained from TD-DFT (B3LYP/LanL2DZ) calculations, the vertical lines correspond to the vertical excitation energies with oscillator strength greater than 0.006 and were expressed as Gaussian functions to generate the spectrum (half-width at half maximum 3000 cm^{-1}).

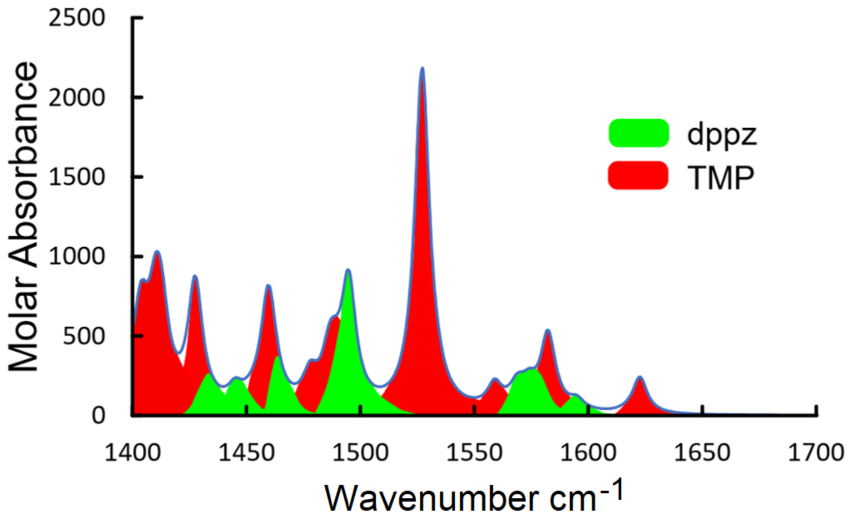

Figure S9. Simulated IR spectra indicating the origin of the vibrations in $[^4Cr(TMP)_2dppz]^{3+}$. Calculations were performed using a B3LYP hybrid DFT coupled to the double zeta quality LanL2DZ basis set. Harmonic spectra have been uniformly scaled by 0.973.

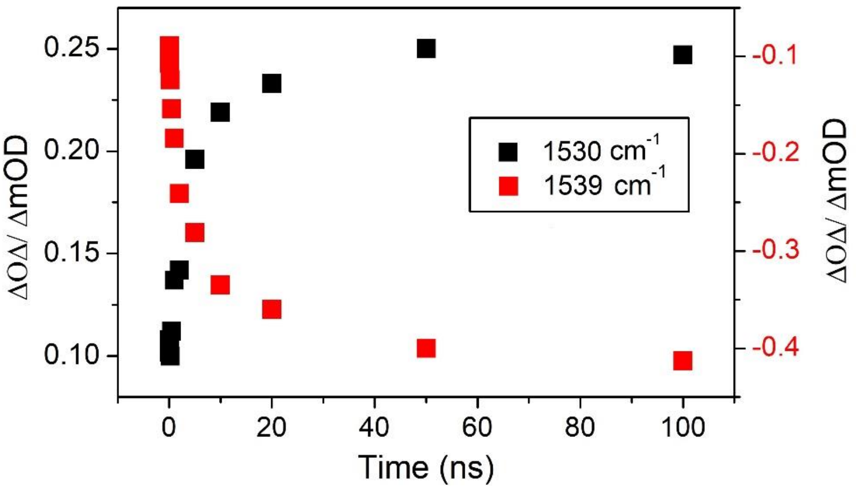

Figure S10. Kinetic analysis of the ps-TRIR spectrum of 1 mM of $\mathbf{[Cr(TMP)_2dppz]^{3+}}$ in presence of 50 mM of sodium phosphate buffer in D_2O monitoring the grow in of the transient band at 1530 cm^{-1} and the bleach band at 1539 cm^{-1} after 400 nm excitation.

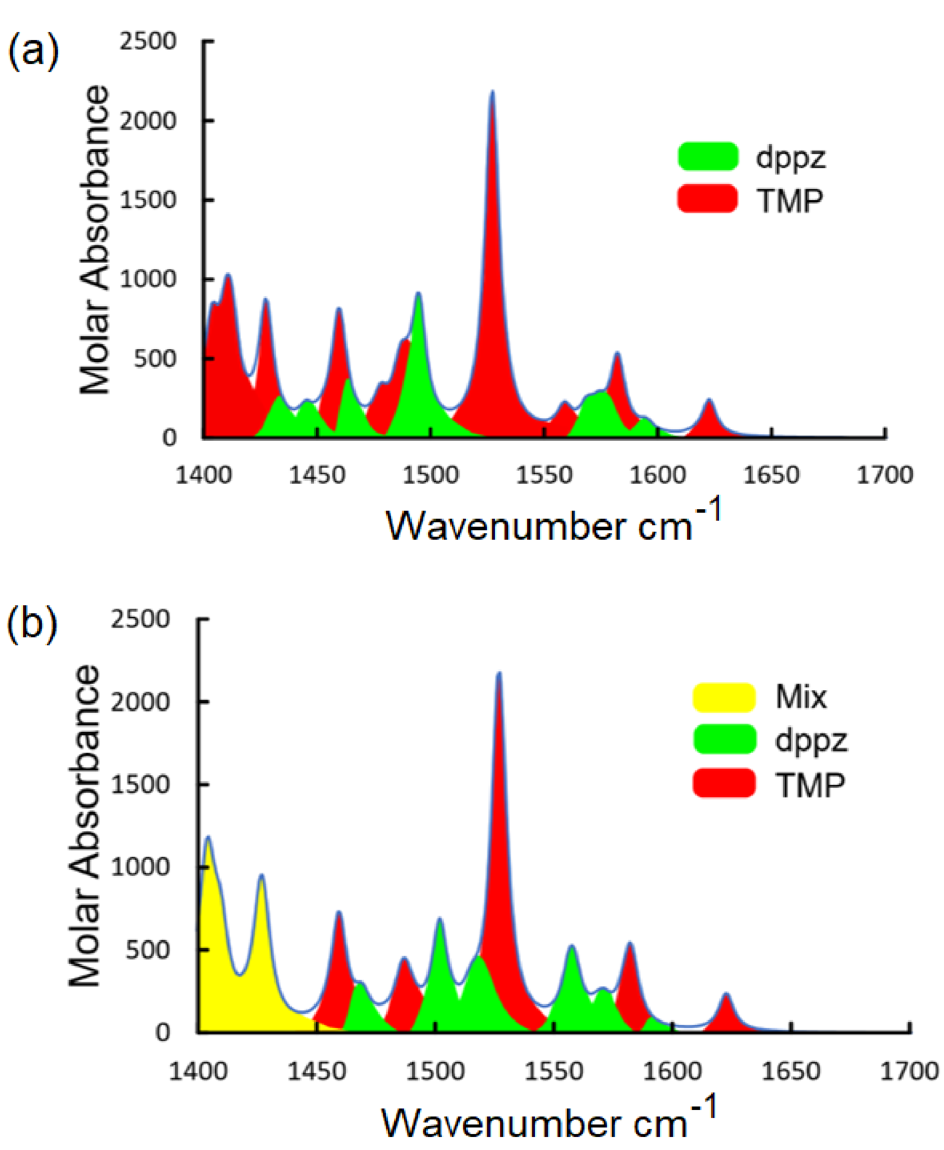

Figure S11. Comparison of the simulated IR spectra indicating the origin of the vibrations in (a) $[^4Cr(TMP)_2dppz]^{3+}$ and (b) $[^4Cr(TMP)_2{}^3dppz]^{3+*}$. Calculations were performed using a B3LYP hybrid DFT coupled to the double zeta quality LanL2DZ basis set. Harmonic spectra have been uniformly scaled by 0.973.

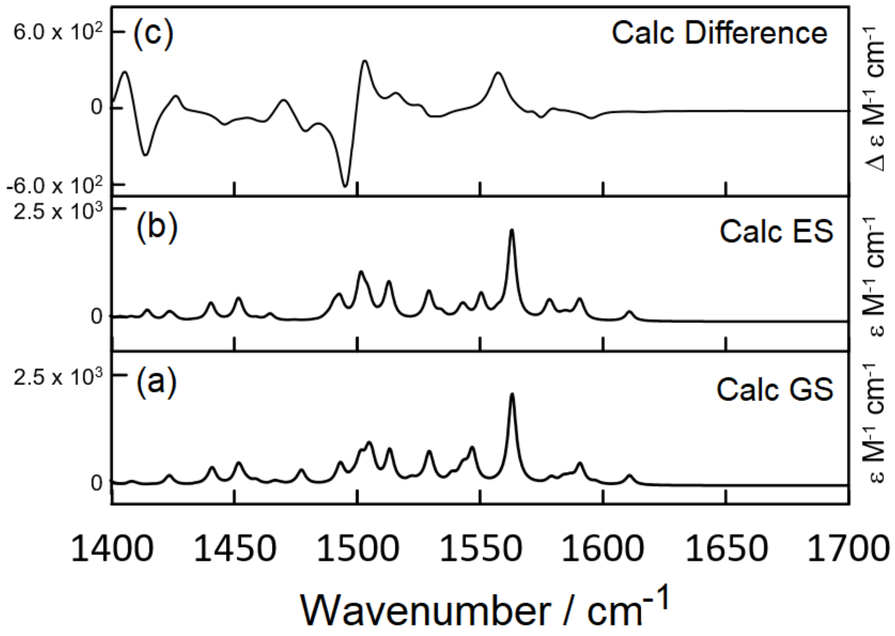

Figure S12. (a) The simulated IR spectrum of the quartet $[^4Cr(TMP)_2dppz]^{3+}$ complex in water; (b) the simulated IR spectrum of the $[^4Cr(TMP)_2{}^3dppz]^{3+*}$ species and (c) the resulting difference spectrum ((b) minus (a)) simulating the difference spectrum obtained from TRIR experiments. Calculations were performed using a B3LYP hybrid DFT coupled to the double zeta quality LanL2DZ basis set. Harmonic spectra have been uniformly scaled by 0.973.

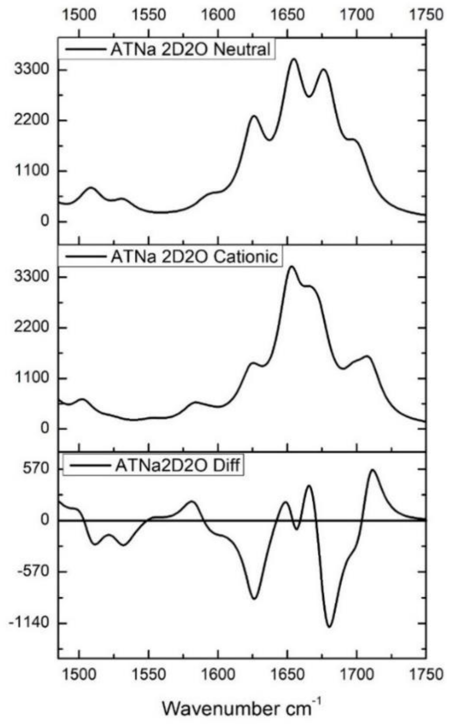

Figure S13. Computed difference spectra between the cationic and the neutral forms of the ATNa.$2D_2O$ model. PCM/M052X/6-31G(d) calculations. On the abscissae frequency (in cm^{-1}). On the ordinate, intensities D (10-40 esu^2 cm^2). Each transition has been broadened with a Gaussian with h.w.h.m=10 cm^{-1}.

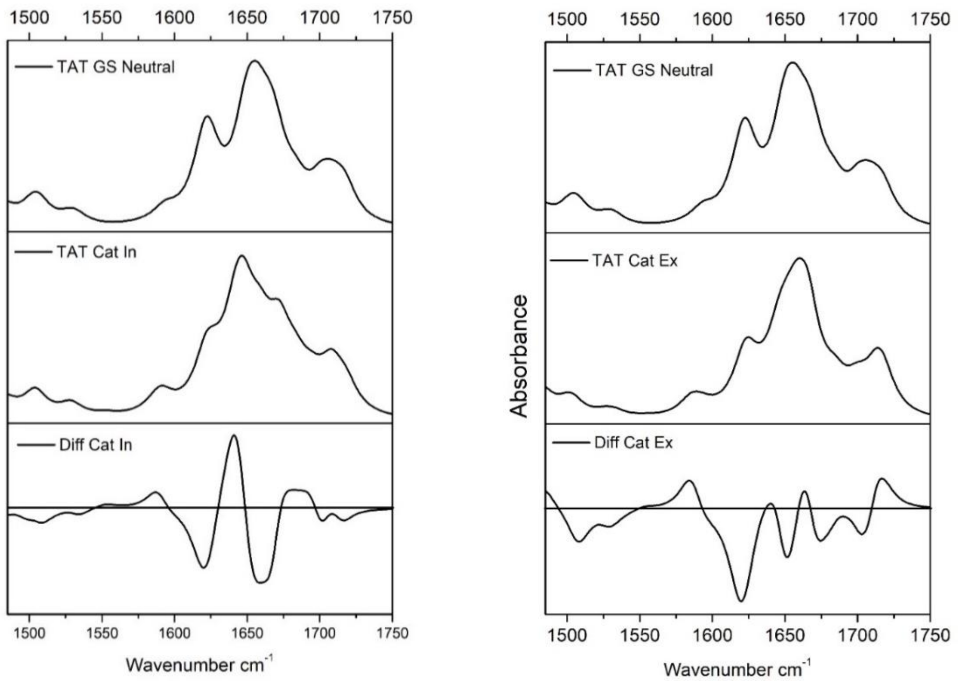

Figure S14. Computed difference spectra between the cationic and the neutral forms of the different TAT models, (Left) Internal Ade^+ cation and (right) termina/external Ade^+ cation. PCM/M052X/6-31G(d) calculations. On the abscissae frequency (in cm-1). On the ordinate, intensities D (10-40 esu^2 cm^2). Each transition has been broadened with a Gaussian with h.w.h.m=10 cm^{-1}.

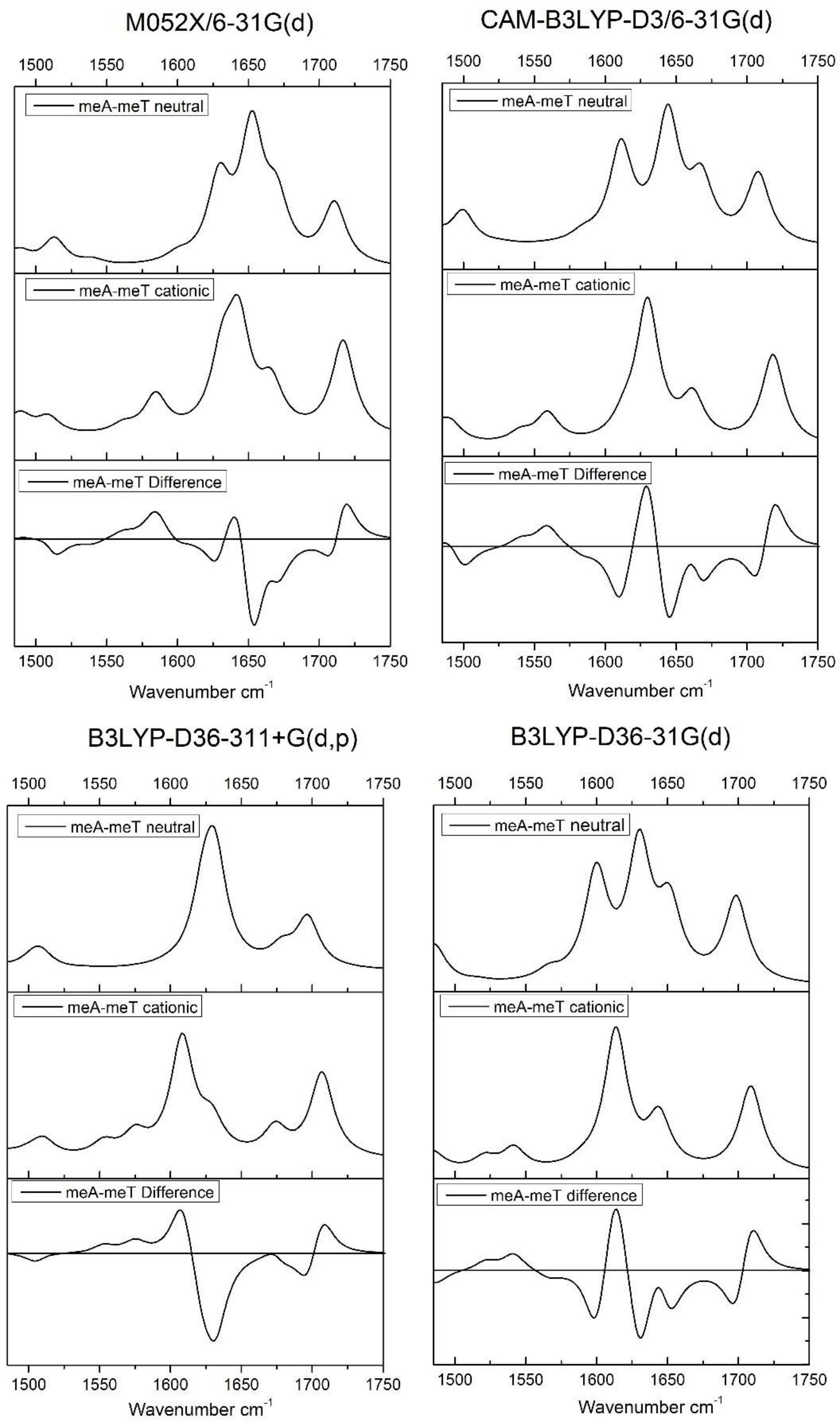

Figure S15. Computed difference spectra between the cationic and the neutral forms of the simple 9methylAdenine-1methylThymine WC dimer calculated using M052X/6-31G(d), B3LYP-D3 (both with 6-31G(d) and 6-311+G(d,p) basis set) and CAM-B3LYP-D3 (with the 6-31G(d) basis set) as indicated. All PCM/M052X/6-31G(d) calculations. On the abscissae frequency (in cm-1). On the ordinate, intensities D (10-40 esu^2 cm^2). Each transition has been broadened with a Gaussian with h.w.h.m=10 cm^{-1}.

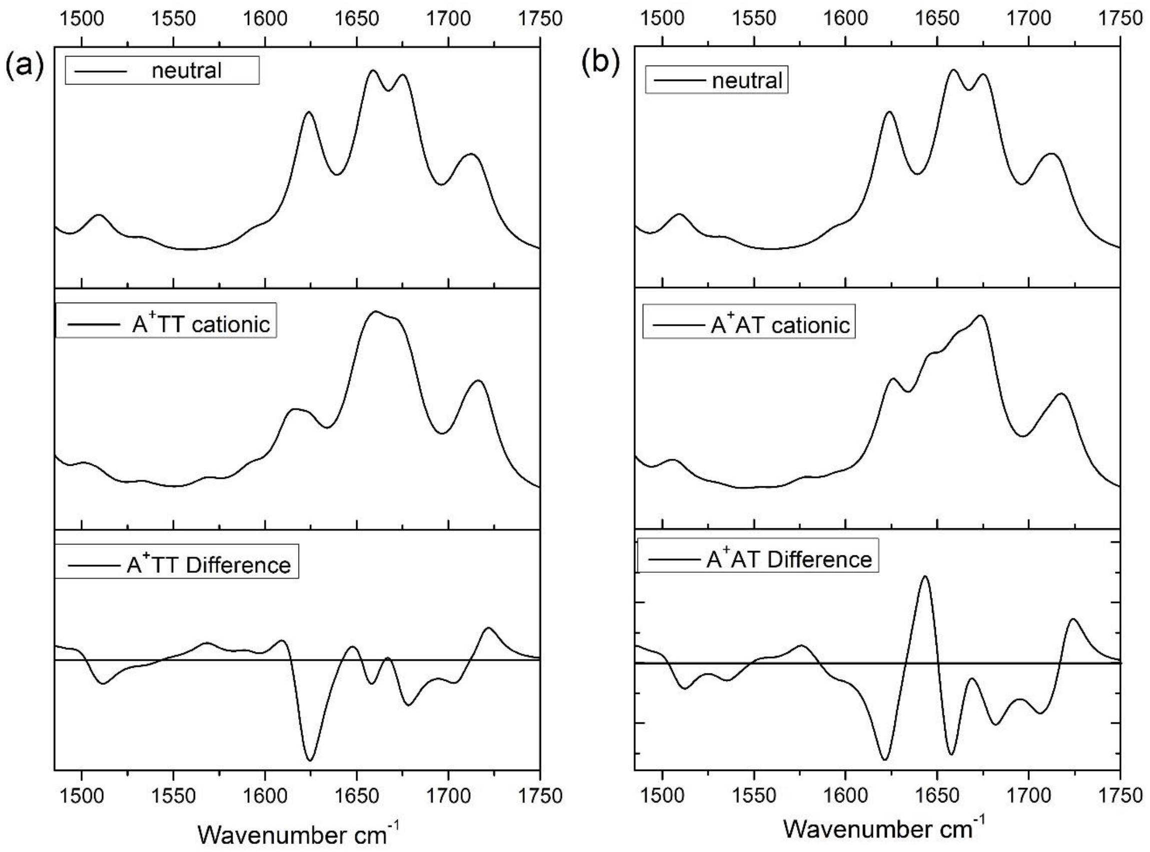

Figure S16. Computed difference spectra between the cationic and the neutral forms of the Watson Crick base paired AAT trimer model system where Ade$^+$ cation located in (a) the AAT step and (b) the ATT step. All PCM/M052X/6-31G(d) calculations. On the abscissae frequency (in cm-1). On the ordinate, intensities D (10-40 esu^2 cm^2). Each transition has been broadened with a Gaussian with h.w.h.m=10 cm^{-1}.

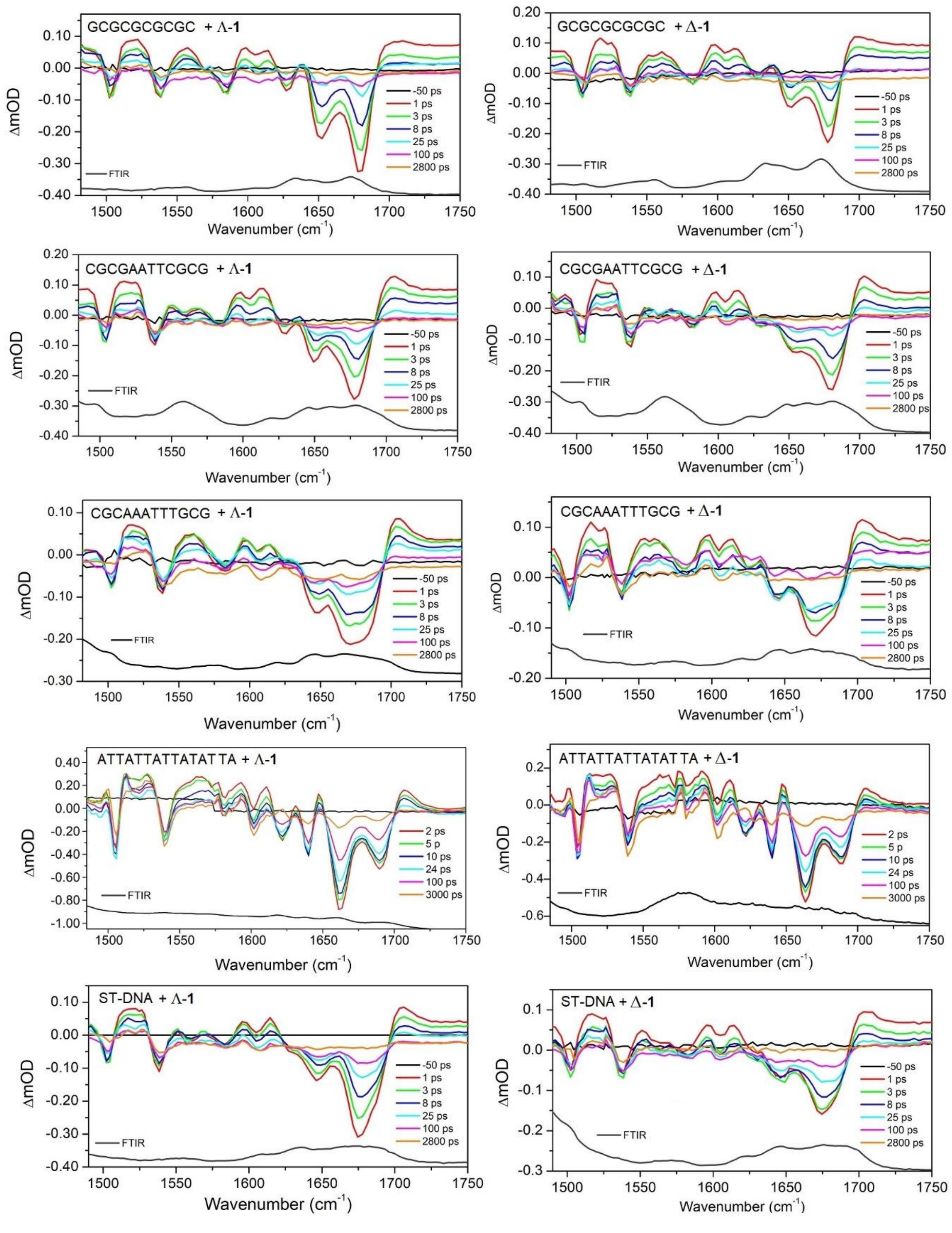

Figure S17. ps-TRIR spectra of 1 mM Λ-1 and Δ-1 in the presence of different DNA systems in 50 mM of potassium phosphate buffer in D_2O after 373 nm excitation (with 400 nm excitation for ATTATTATTATATTA).

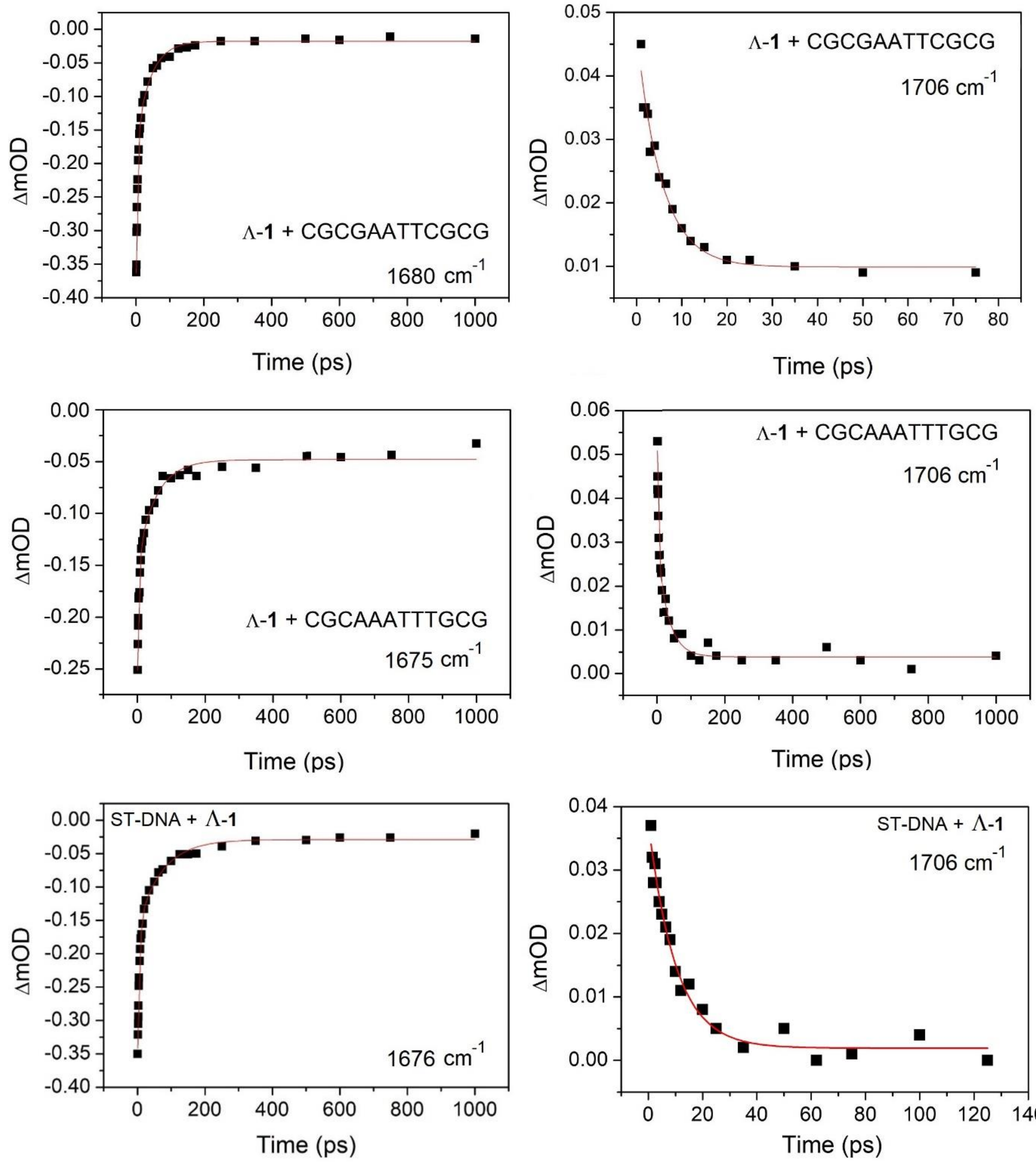

Figure S18. Kinetic analysis of the ps-TRIR spectra of 1 mM of **Λ-[Cr(TMP)$_2$dppz]$^{3+}$** in presence of 25 mM nucleotide of oligonucleotide systems in 50 mM of potassium phosphate buffer in D_2O upon excitation at 373 nm monitored at the carbonyl bleach and transient band. Given in Table 1.

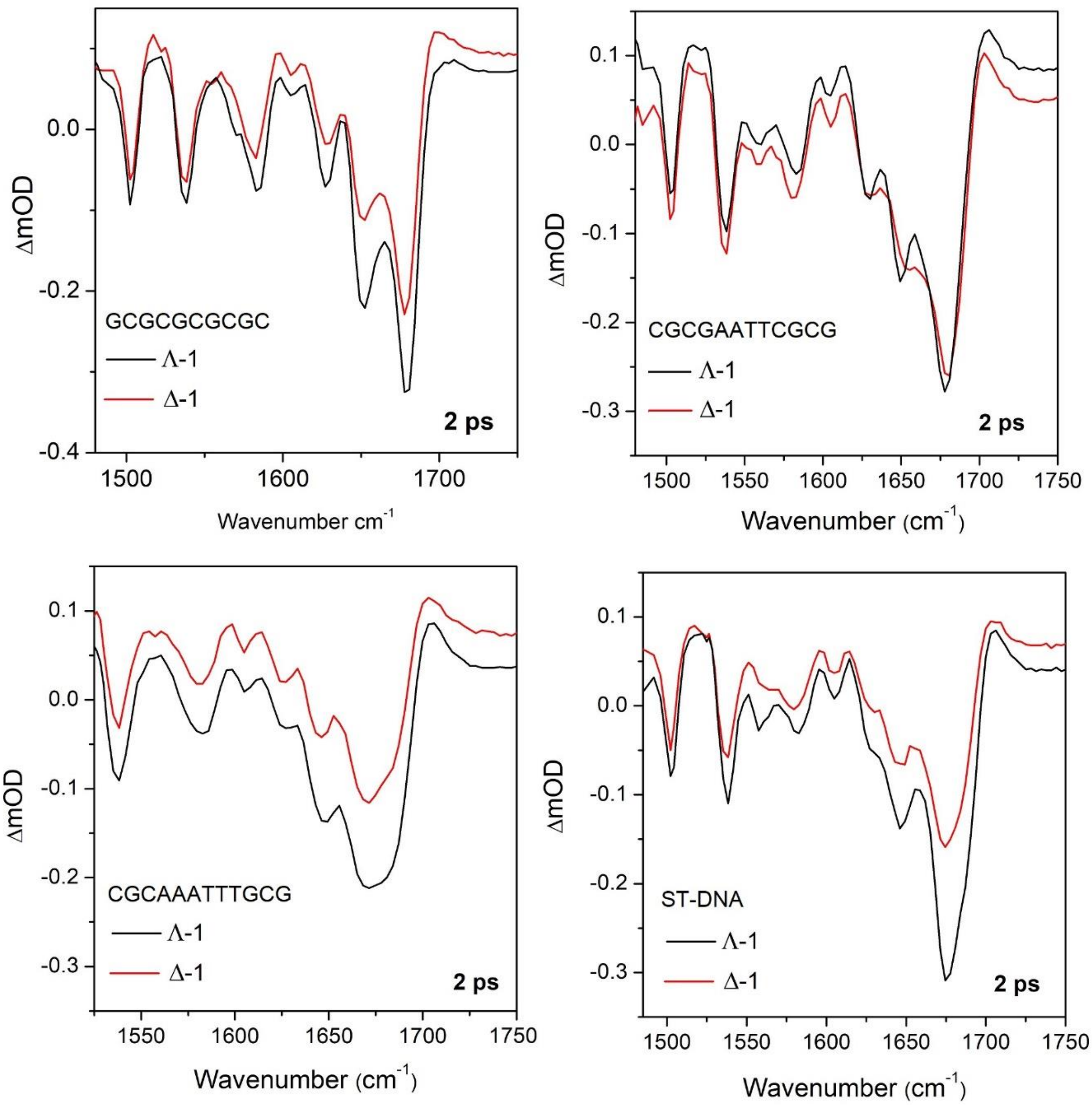

Figure S19. ps-TRIR spectra of 1 mM Λ-1 and Δ-1 in the presence of various GC-containing oligodeoxynucleotide systems in 50 mM of potassium phosphate buffer in D_2O after 373 nm excitation. *Not adjusted to match baseline offset.

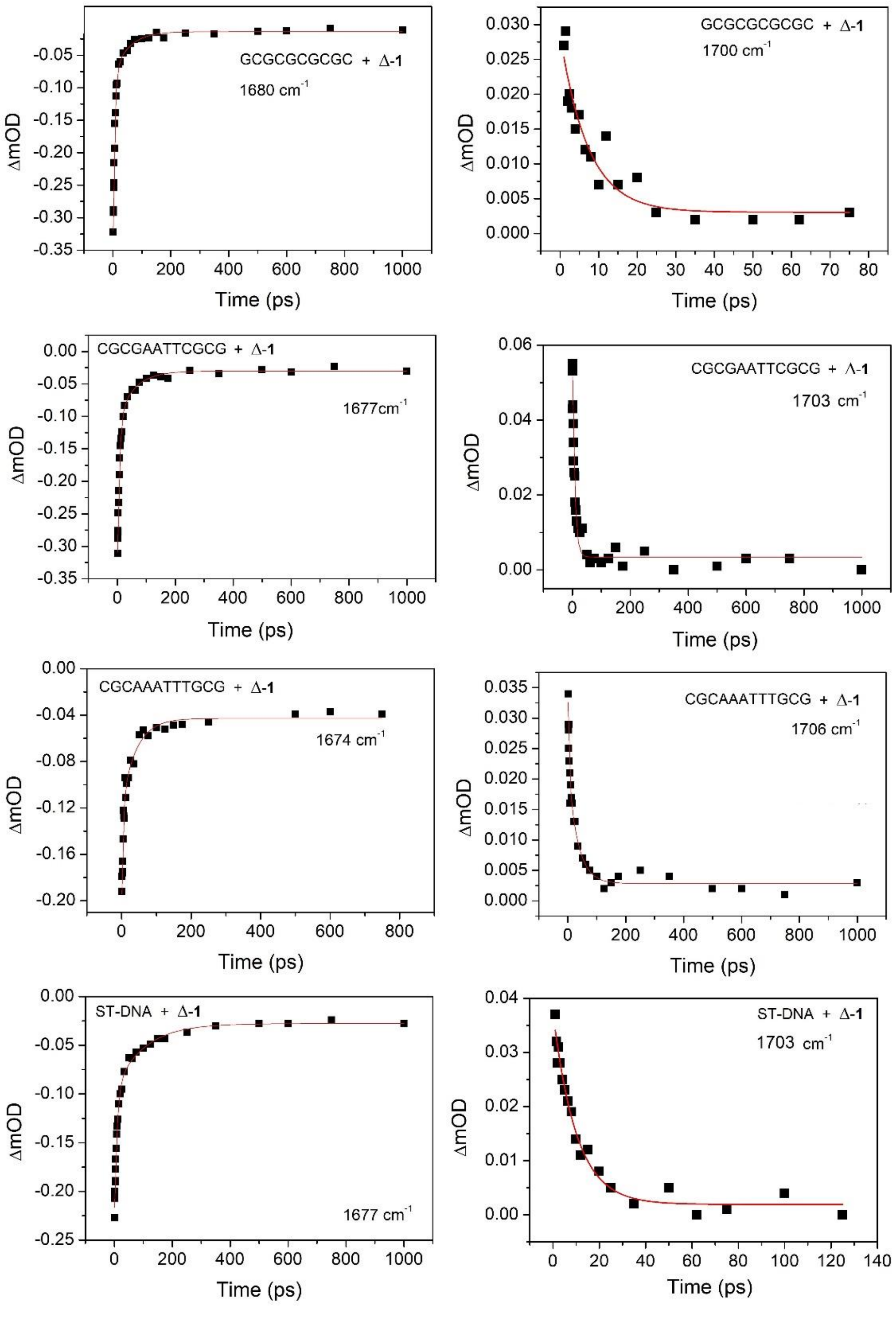

Figure S20. Kinetic analysis of the ps-TRIR spectra of 1 mM of **Δ-[Cr(TMP)$_2$dppz]$^{3+}$** in presence of 25 mM nucleotide of oligonucleotide systems in 50 mM of potassium phosphate buffer in D_2O upon excitation at 373 nm monitored at the carbonyl bleach and transient band. Given in Table 1.

Section S3 Tables

Table S1. CD spectroscopy data of enantiomers of 1 acetonitrile. Cell pathlength = 1 cm.

Enantiomer	$\Delta\varepsilon$ (260 nm) M^{-1} cm^{-1}	$\Delta\varepsilon$ (280 nm) M^{-1} cm^{-1}	$\Delta\varepsilon$ (345 nm) M^{-1} cm^{-1}	Optical Enhancement % present*
Δ	+41.5	-80.0	-19.6	76.5 %
Λ	-44.6	+89.4	+24.4	79.0 %

*Based on $\Delta\varepsilon_{280\ nm}$ (± 155 M^{-1} cm^{-1}) for the Λ and Δ–**[Cr(TMP)$_2$dppz]$^{3+}$** enantiomers.[12]

Table S2. Binding constants of lambda and delta enantiomers in presence of **DNA systems** in presence of 50 mM potassium phosphate buffer (Emission data).

**System**	***% quenched***	**K_{DNA} (M^{-1})**	**Binding Site Size, *n* (base pairs)**
**Λ-(GC)$_5$**	94	7.7×10^5	3.1 ± 0.1
**Δ-(GC)$_5$**	91	6.1×10^5	4.8 ± 0.2
**Λ-CGCGAATTCGCG**	98	1.4×10^6	1.6 ± 0.1
**Δ-CGCGAATTCGCG**	96	5.1×10^5	1.7 ± 0.1
**Λ-CGCAAATTTGCG**	97	1.7×10^6	1.92 ± 0.04
**Δ- CGCAAATTTGCG**	95	4.8×10^5	2.10 ± 0.09
**Λ-ATTATTATTATATT**	64	$4.0 \text{ x } 10^6$	2.25 ± 0.05
**Δ-ATTATTATTATATT**	62	$3.0 \text{ x } 10^6$	2.28 ± 0.05
**Λ-salmon sperm**	94	1.4×10^6	3.20 ± 0.03
**Δ-salmon sperm**	94	7.1×10^5	2.74 ± 0.02

Table S3. Assignment of infrared bands of **1** by DFT.

ν(cm^{-1})	Normal Mode	Displacement Vector Location	ν(cm^{-1})	Normal Mode	Displacement Vector Location
1622	255	TMP	1459.53	223	TMP
			1549.52	222	TMP
1581	249	TMP	1459.2	221	TMP
			1459.1	220	TMP
1527	241	TMP			
			1427	212	TMP
1494	238	dppz			
			1410	207	TMP
			1404	206	TMP

Table S4. Assignment of infrared bands of **AT** ODN fragment by DFT.[a] [scaled values in parenthesis]

ῡ (cm^{-1})	Assignment
1780;1781 [1700;1701]	Thymine, symmetric stretching C=O (predominant contribution from the C2=O2 bond)
1759;1758 [1679;1678]	Thymine, C5=C6 stretching mode
1733;1730 [1655;1652]	Thymine: anti-symmetric stretching C=O (predominantly C4=O4 bond involved in the WC HB)
1696; 1698 [1619;1621]	Adenine ring stretching

[a] The presence of strong Hydrogen Bond (HB) and stacking interactions leads to a mixing between the vibrational motions of the nucleobases, which, in some cases, can be significant.

Section S4 References

1. Carter, M. T.; Rodriguez, M.; Bard, A. J., Voltammetric studies of the interaction of metal chelates with DNA. 2. Tris-chelated complexes of cobalt(III) and iron(II) with 1,10-phenanthroline and 2,2'-bipyridine. *J. Am. Chem. Soc.* **1989,** *111*, 8901-8911.
2. Frisch, M. J.; Trucks, G. W.; Schlegel, H. B.; Scuseria, G. E.; Robb, M. A.; Cheeseman, J. R.; Scalmani, G.; Barone, V.; Petersson, G. A.; Nakatsuji, H.; Li, X.; Caricato, M.; Marenich, A. V.; Bloino, J.; Janesko, B. G.; Gomperts, R.; Mennucci, B.; Hratchian, H. P.; Ortiz, J. V.; Izmaylov, A. F.; Sonnenberg, J. L.; Williams-Young, D.; Ding, F.; Lipparini, F.; Egidi, F.; Goings, J.; Peng, B.; Petrone, A.; Henderson, T.; Ranasinghe, D.; Zakrzewski, V. G.; Gao, J.; Rega, N.; Zheng, G.; Liang, W.; Hada, M.; Ehara, M.; Toyota, K.; Fukuda, R.; Hasegawa, J.; Ishida, M.; Nakajima, T.; Honda, Y.; Kitao, O.; Nakai, H.; Vreven, T.; Throssell, K.; Montgomery, J. A.; Peralta, J. E.; Ogliaro, F.; Bearpark, M. J.; Heyd, J. J.; Brothers, E. N.; Kudin, K. N.; Staroverov, V. N.; Keith, T. A.; Kobayashi, R.; Normand, J.; Raghavachari, K.; Rendell, A. P.; Burant, J. C.; Iyengar, S. S.; Tomasi, J.; Cossi, M.; Millam, J. M.; Klene, M.; Adamo, C.; Cammi, R.; Ochterski, J. W.; Martin, R. L.; Morokuma, K.; Farkas, O.; Foresman, J. B.; Fox, D. J. *Gaussian 16*, Revision B.01; Gaussian, Inc., : Wallingford CT, 2016.
3. Dennington II, R. D.; Keith, T. A.; Millam, J. M. *GaussView*, Semichem, Inc.: 2016.
4. Gorelsky, S. I. *AOMix Program for Molecular Orbital Analysis* http://www.sg-chem.net/, version 6.88; University of Ottawa: Ottawa, 2013.
5. Gorelsky, S. I.; Lever, A. B. P., Electronic structure and spectra of ruthenium diimine complexes by density functional theory and INDO/S. Comparison of the two methods. *J. Organomet. Chem.* **2001,** *635*, 187-196.
6. Becke, A. D., Density-Functional Thermochemistry 3. The Role of Exact Exchange. *J. Chem. Phys.* **1993,** *98*, 5648-5652.
7. Lee, C. T.; Yang, W. T.; Parr, R. G., Development of the Colle-Salvetti Correlation-Energy Formula into a Functional of the Electron-Density. *Phys. Rev. B* **1988,** *37*, 785-789.
8. Dunning Jr, T. H.; Hay, P. J., Modern Theoretical Chemistry. In *Modern Theoretical Chemistry*, Schaefer III, H. F., Ed. Plenum Press: New York, 1977; Vol. 3.
9. Tomasi, J.; Mennucci, B.; Cammi, R., Quantum mechanical continuum solvation models. *Chem. Rev.* **2005,** *105*, 2999-3093.
10. Keane, P. M.; Tory, J.; Towrie, M.; Sazanovich, I. V.; Cardin, C. J.; Quinn, S. J.; Hartl, F.; Kelly, J. M.; Long, C., Spectro-electrochemical Studies on $[Ru(TAP)_2(dppz)]^{2+}$ - Insights into the Mechanism of its Photosensitized Oxidation of Oligonucleotides. *Inorg. Chem.* **2019**.
11. Poynton, F. E.; Hall, J. P.; Keane, P. M.; Schwarz, C.; Sazanovich, I. V.; Towrie, M.; Gunnlaugsson, T.; Cardin, C. J.; Cardin, D. J.; Quinn, S. J.; Long, C.; Kelly, J. M., Direct observation by time-resolved infrared spectroscopy of the bright and the dark excited states of the $[Ru(phen)_2(dppz)]^{2+}$ light-switch compound in solution and when bound to DNA. *Chem. Sci.* **2016,** *7*, 3075-3084.
12. Vandiver, M. S.; Bridges, E. P.; Koon, R. L.; Kinnaird, A. N.; Glaeser, J. W.; Campbell, J. F.; Priedemann, C. J.; Rosenblatt, W. T.; Herbert, B. J.; Wheeler, S. K.; Wheeler, J. F.; Kane-Maguire, N. A., Effect of ancillary ligands on the DNA interaction of $[Cr(diimine)_3]^{3+}$ complexes containing the intercalating dipyridophenazine ligand. *Inorg. Chem.* **2010,** *49*, 839-48.
